# Efficacy and safety of antibody-drug conjugate based therapy in locally advanced or metastatic urothelial carcinoma: a systematic review and network meta-analysis of emerging clinical evidence

**DOI:** 10.3389/fimmu.2026.1728521

**Published:** 2026-03-24

**Authors:** Youran Dai, Chenwei Xiao, Liang Wang, Wenguang Zhou, Ruiqing Bo, Zerun Cheng, Guofeng Pan

**Affiliations:** 1 Department of Traditional Chinese Medicine, Beijing Shijitan Hospital affiliated to Capital Medical University, Beijing, China; 2 The First Affiliated Hospital of Zhejiang Chinese Medical University (Zhejiang Provincial Hospital of Chinese Medicine), Hangzhou, Zhejiang, China; 3 Department of Andrology, Xiyuan Hospital, China Academy of Chinese Medical Sciences, Beijing, China; 4 Qihuang Chinese Medicine school, Beijing University of Chinese Medical, Beijing, China

**Keywords:** ADC, antibody-drug conjugate, meta-analysis, systematic review, urothelial carcinoma

## Abstract

**Introduction:**

Locally advanced or metastatic urothelial carcinoma (la/mUC) is associated with poor prognosis and limited treatment options. Antibody-drug conjugates (ADCs) have emerged as a promising therapeutic approach. While previous meta-analyses have shown the efficacy and safety of ADCs in UC, the rapid development of new ADC agents and combination therapies necessitates an updated and comprehensive evidence synthesis.

**Materials and methods:**

A comprehensive search was performed in PubMed, Embase, Cochrane Library, and Web of Science from inception to September 2025. Interested outcomes include overall survival (OS), progression-free survival (PFS), objective response rate (ORR), disease control rate (DCR), and adverse events (AES). RoB 2.0, ROBINS-I tools and GRADE framework were used for quality assessment.

**Results:**

A total of 30 studies (5 RCTs and 25 single-arm trails), involving 3,631 patients, were included. Network meta-analysis showed that, compared with standard therapy, enfortumab vedotin (EV) in combination with pembrolizumab significantly improved OS (HR = 0.63, 95%CI: 0.43-0.92), ORR (OR = 3.33, 95% CI: 1.65-6.74), and PFS (HR = 0.48, 95% CI: 0.41-0.57). The safety results indicate that the ADC agents have a higher incidence rate of ≥3AES. The analysis of single-arm trails revealed that bulumtatug fuvedotin combined with toripalimab achieved an ORR as high as 88% (95% CI: 73%-96%), while disitamab vedotin-based combination therapy showed the longest median OS.

**Conclusions:**

This study provides a comprehensive synthesis of the latest clinical evidence on ADC-based monotherapy and combination regimens for la/mUC. The findings confirm the compelling efficacy and manageable safety profile of ADCs in this setting, while also underscoring the need for further clinical trials to validate and refine personalized treatment strategies.

**Systematic Review Registration:**

https://www.crd.york.ac.uk/PROSPERO/, identifier CRD 420251114022.

## 1 Introduction

Globally, urological cancers account for approximately 13% of all cancer cases, posing a substantial public health burden (1, 2). Among these, bladder cancer ranks as the ninth most frequently diagnosed malignancy. Urothelial carcinoma (UC) is the predominant histological subtype, comprising over 90% of all cases, with the highest incidence rates observed in Europe and the United States (U.S.) (1). It is estimated that in 2025, U.S. will see approximately 84,870 new cases and 17,420 deaths from this disease (3). Locally advanced or metastatic UC (la/mUC) is characterized by its aggressive nature and high recurrence rate, leading to poor overall survival (OS). Despite significant therapeutic advances, systemic treatment remains challenging. Conventional treatments for UC, including platinum-based chemotherapy and immune checkpoint inhibitors (ICI), often yield suboptimal outcomes.

ADCs are typically composed of a monoclonal antibody covalently linked to a cytotoxic agent via a chemical linker (4). By combining the high target specificity of antibodies with the potent cell-killing activity of cytotoxic drugs, ADCs enable precise and efficient elimination of cancer cells. Characterized by an improved therapeutic index and reduced systemic toxicity, ADCs have emerged as a key component of precision oncology and represent a promising treatment strategy (5). Consequently, there has been growing interest in the application of antibody-drug conjugates (ADCs) (6, 7). Up to now, two ADCs, enfortumab vedotin (EV) and sacituzumab govitecan (SG), have received full or accelerated approval from the U.S. Food and Drug Administration for the treatment of UC (8). These ADCs provide new therapeutic options for patients with UC, filling a critical gap in the management of progressive disease. Additionally, combination therapies of ADCs and other drugs are under active investigation. Previous studies have established the value of ADCs in la/mUC (9), and recent systematic reviews have further explored their role in urological cancers (10, 11). However, the rapidly evolving treatment landscape has outpaced these evidence syntheses. With the recent reporting of pivotal Phase III trials such as EV-302, and the continuous emergence of novel ADCs (e.g., MRG002, 9MW2821) and their combinations with ICIs, prior analyses no longer adequately reflect current clinical advances. Notably, most studies are limited to monotherapies or specific drug combinations, lacking stratified comparisons of efficacy and safety across different ADC regimens. There also remains a gap in the systematic integration of randomized controlled trials (RCTs) with single-arm trial and real-world evidence. Therefore, an updated and comprehensive synthesis is urgently needed to consolidate the rapidly accumulating clinical data, clarify the value ordering of various ADC treatment options, and inform both clinical decision-making and future research directions.

This study aims to systematically synthesize and analyze emerging clinical evidence, including data from RCTs, single-arm studies, and real-world sources. We aim to conduct a stratified evaluation of the efficacy and safety profiles across the spectrum of ADC-based regimens, including both novel monotherapies and combination strategies in la/mUC. This comprehensive methodological approach will generate robust comparative evidence that not only offers novel insights into the clinical application of ADCs and expands available treatment options, but also helps establish a hierarchical understanding of the rapidly evolving ADC therapeutic landscape.

## 2 Materials and methods

This systematic review and meta-analysis were conducted in accordance with the recommendations outlined in the Cochrane Handbook for Systematic Reviews of Interventions and adhered to the Preferred Reporting Items for Systematic Reviews and Meta-Analyses (PRISMA) 2020 guidelines (12). The protocol of this study was registered in PROSPERO (CRD 420251114022).

### 2.1 Search strategy

A comprehensive literature search was conducted across various electronic databases, including Embase, the Cochrane Library, PubMed, and Web of Science, span from the inception of each database to September, 2025. We also reviewed abstract archives from major oncology conferences, including those organized by the European Society for Medical Oncology (ESMO) and the American Society of Clinical Oncology (ASCO). The search strategy incorporated both Medical Subject Headings (MeSH) terms and free-text words, centered on key concepts such as “transitional cell carcinoma” and “antibody drug conjugate.” Additional search terms were applied to identify approved or investigational ADCs used in the treatment of UC. ADCs of interest included, but were not limited to: Enfortumab Vedotin, Sacituzumab Govitecan, Trastuzumab Deruxtecan, Disitamab Vedotin, Telisotuzumab Vedotin, Mirvetuximab Soravtansine, Trastuzumab Vedotin, MGR002, Bulumtatug Fuvedotin, Zelenectide pevedotin, 9MW2821, BT8009, A166, ARX788, ASG-15ME, ASG-22ME and DS-8201. To ensure comprehensive coverage, we manually screened the reference lists of all included studies and relevant review articles. The full search strategy is available in **Supplementary Table 1**.

### 2.2 Inclusion and exclusion criteria

Studies were included based on the following eligibility criteria: a) original articles (RCTs or single-arm trials); b) adult patients (≥18 years) with a confirmed diagnosis of UC, without severe hepatic or renal dysfunction; c) ADC monotherapy or ADC-based combinations therapy; d) have reported at least one of the following outcomes: OS, objective response rate (ORR), progression-free survival (PFS), disease control rate (DCR), and grade ≥3 adverse events (AEs). No restrictions had been applied regarding race, ethnicity, sex, tumor stage or molecule. The exclusion criteria included: a) animal studies, reviews, letters, study protocols, case reports, and duplicate publications; b) non-urothelial malignancies; c) studies that did not report any interested outcomes; d) not published in English. To capture the most current evidence, conference abstracts that reported outcomes of interest with extractable data were considered for inclusion. For studies identified only through abstracts, we conducted supplementary manual searches to verify whether a corresponding full-text publication had subsequently appeared. In all cases, the full-text article was used if available; otherwise, the meeting abstract was retained as the data source.

### 2.3 Outcomes

The primary outcome was OS, defined as the time from randomization to death from any cause. OS is regarded as the optimal endpoint for evaluating the efficacy of oncological therapies (13, 14). Secondary outcomes included ORR, PFS, DCR and grade ≥3 AEs.

### 2.4 Study selection

Two researchers independently performed the literature search using the specified databases. All retrieved records were imported into EndNote 21 (Clarivate Analytics) for reference management and screening. The initial screening phase involved a blinded review of titles and abstracts by both investigators in accordance with the predefined eligibility criteria. Subsequently, the full texts of the remaining potentially eligible articles were independently assessed by two researchers for final inclusion. Any disagreements encountered during the screening or full-text evaluation stages were resolved through consensus discussion or, when required, by consultation with a senior investigator.

### 2.5 Data extraction

Two researchers (YD and CX) independently extracted data using a standardized form. The following details were collected from each included study: first author, country, year of publication, trial name, sample size, gender proportion, median age, median follow-up duration, treatment regimens, reported endpoints, and other relevant variables required for analysis. Key efficacy and safety outcomes consisted of OS, ORR, PFS, DCR, and grade ≥3 AEs. For RCTs, when survival data were presented only as Kaplan-Meier curves, we used Engauge Digitizer software (version 12.1) following the method described by Tierney et al. (15) to extract numerical values and subsequently estimate HRs (16). Multi-arm trials were treated as separate two-arm comparisons. Data from RCTs and single-arm studies were extracted separately. Any discrepancies between investigators during data extraction were resolved through discussion or, when needed, by arbitration from a third reviewer (GP).

### 2.6 Risk of bias assessment

The risk of bias was assessed using two validated tools: the Revised Cochrane Risk of Bias tool (Version 2, RoB 2.0) (17) for RCTs, and the Risk of Bias in Non-randomized Studies of Interventions (ROBINS-I) tool (18) for single-arm studies. The ROB 2.0 tool evaluates bias across five domains: the randomization process, deviations from intended interventions, missing outcome data, measurement of the outcome, and selection of the reported result. The ROBINS-I tool covers seven domains of bias: confounding, selection of participants, classification of interventions, deviations from intended interventions, missing data, measurement of outcomes, and selection of the reported result. Each domain was judged as “low risk”, “some concerns”, or “high risk.” The Grading of Recommendations Assessment, Development, and Evaluation (GRADE) framework was used to assess the overall certainty of evidence (19). Following Cochrane methods, summary of assessments were generated with GRADEpro software. The certainty for each comparison was rated as high, moderate, low, or very low, based on evaluations of study design, risk of bias, inconsistency, indirectness, imprecision, and other applicable considerations. Two investigators (YD and LW) independently performed the assessments for all included studies. Disagreements were resolved through discussion with a third reviewer (GP), who provided adjudication and methodological oversight.

### 2.7 Statistical analysis

All statistical analyses were performed using R (version 4.4.2) and Stata (version 17.0). For RCTs, network meta-analysis (NMA) was conducted using “netmeta” package (20). Empirical and simulation studies have indicated that frequentist and Bayesian approaches generally yield overlapping results in NMA (21). Dichotomous outcomes, including ORR, DCR, and AEs, were analyzed using odds ratios (ORs) with 95% confidence intervals (CIs). Survival outcomes, including PFS and OS, were summarized using hazard ratios (HRs) with 95% CIs. Network estimates were presented using forest plots and ranking plots, with interventions ranked by P-score. For OS, ORR, PFS, and DCR, a larger P-score indicates a more favorable outcome, while for AES, a smaller P-score is preferred. For networks containing closed loops, local inconsistency was examined via the node-splitting method, which compares direct evidence for a specific treatment contrast against the indirect evidence derived from the remainder of the network. Statistical significance had been defined by 95% CIs excluding the 1 value. We also performed a Bayesian regression analysis using the “gemtc” package, running a Markov chain Monte Carlo simulation with 5,000 adaptation and 20,000 posterior sampling iterations (22), and adjusting for prespecified covariates (mean age, prior therapy, and follow-up duration) where data allowed. To assess the robustness of the primary NMA outcomes, we did two sensitivity analyses excluding studies with small simple size (any group with less than 100 participants), and control arms did not receive treatment of physician’s choice (TPC: paclitaxel 175 mg/m2, docetaxel 75 mg/m2, or vinflunine 320mg/m2, IV, d1, q21d). For single-arm studies, meta-analyses were performed using the “meta” and “metafor” packages. Given the absence of control group, ORR, DCR and AEs were synthesized as pooled proportions (P-pooled) with 95% CIs. Regarding time-to-event endpoints, the reported mPFS and mOS was extracted for analysis. Generalized linear mixed models (GLMMs) were utilized in the analysis. To explore potential sources of heterogeneity, we performed subgroup and regression analyses based on predefined factors such as treatment regimen, tumor molecular subtype, median age, prior therapies, follow-up time, and country, where data availability allowed. To evaluate the robustness of the results, a leave-one-out sensitivity analysis was applied. Both fixed-effect and random-effects models were applied in all meta-analyses. Statistical heterogeneity was evaluated using Cochran’s Q test and the I² statistic, with a Q test *P*-value<0.05 or I²>50% indicating substantial heterogeneity. In such cases, results from the random-effects model were reported. Where data were unsuitable for meta-analysis, we would provide a descriptive synthesis. Publication bias was assessed using funnel plots and Egger’s test when more than 10 studies were available. A two-sided *P*-value <0.05 was considered statistically significant for all analyses.

## 3 Results

### 3.1 Search results

**Figure 1:**
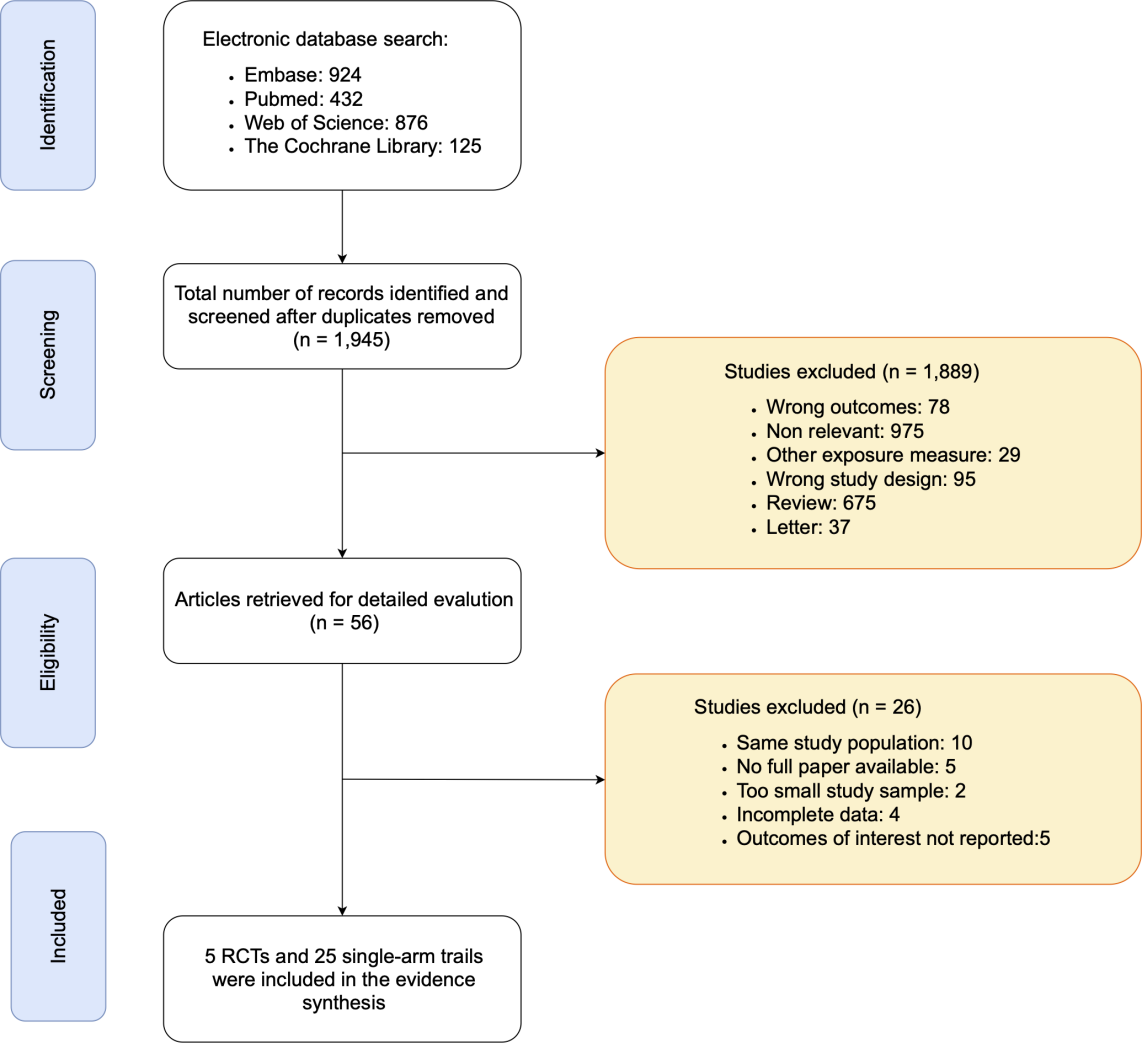
PRISMA flow diagram.

The initial database search identified 2,357 records. After removal of 412 duplicates, 1,945 unique records remained for title and abstract screening. Of these, 1,889 records were excluded due to irrelevance, duplication, or failure to meet the predefined eligibility criteria. The remaining 56 articles underwent full-text review, leading to the exclusion of 26 studies for the following reasons: overlapping patient cohorts, insufficient or unavailable outcome data, small sample size, or incomplete reporting. Ultimately, 5 RCTs (23–27) and 25 single-arm studies (24, 28–52) were included in the final analysis (**Figure 1**).

### 3.2 Basic characteristics of the included literature

A total of 30 studies were included in this study, comprising 5 RCTs, 25 single-arm studies (include 2 real-world study), with an aggregate of 3,631 patients diagnosed with la/mUC. The included studies were conducted across multiple countries, including the United States, United Kingdom, China, France, and Japan. All studies evaluated interventions based on ADCs, either as monotherapy or as the backbone of combination regimens. The ADC agents investigated included EV, disitamab vedotin (DV), SG, trastuzumab vedotin (TV, MRG002), zelenectide pevedotin (ZP), bulumtatug fuvedotin (BF), trastuzumab deruxtecan (T-DXd), and trastuzumab emtansine (T-DM1). 8 studies assessed the efficacy of ADC combine with ICI or ADC-ADC dual therapy. In RCTs, the interventions included were SG, EV, and EV plus pembrolizumab. These therapies were primarily compared with treatment of physician’s choice (TPC: paclitaxel 175 mg/m2, docetaxel 75 mg/m2, or vinflunine 320 mg/m2, IV, d1, q21d). One RCT evaluated EV plus pembrolizumab versus EV monotherapy, thus forming a unified evidence network through EV as the linking node for indirect comparison with TPC. Across the included studies, patients were generally heavily pretreated. The median age ranged from 60 to 72.3 years, and median follow-up duration varied between 7.1 and 33.2 months. Detailed baseline characteristics of the included studies are provided in **Table 1**.

**Table 1:** Baseline characteristics of included studies.

First author	Year	Country	Registration number	Sample size (n/%)		ECOG	Experiment			Control			Prior lines of treatment	Cancer type	Cancer stage	Molecular	Median follow-up time (months)	Study type	Endpoints
				Male	Female		N	Median age (years)	Intervention	N	Median age (years)	Intervention							
T. Powles	2025	British	NCT04527991	563 (79.2%)	148 (20.8%)	0-1	355	67.0 (41-89)	Sacituzumab govitecan 10 mg/kg, d1, d8, q21d	356	68 (30-85)	TPC (paclitaxel 175 mg/m2, docetaxel 75 mg/m2, or vinflunine 320mg/m2), IV, d1, q21d	Platinum-based chemotherapy/anti PD-L1 therapy	aUC	IV	/	9.2	RCT	OS, PFS, ORR
T. Powles	2024	British	NCT04223856	680 (76.7%)	206 (23.3%)	0-2	442	69.0 (37-87)	Enfortumab vedotin 1.25 mg/kg, IV, d1, d8, d5,q28d+pembrolizumab 200 mg d1, q21d	444	69 (22-91)	TPC (paclitaxel 175 mg/m2, docetaxel 75 mg/m2, or vinflunine 320mg/m2), IV, d1, q21d	Previously untreated	la/mUC	IV	/	17.2	RCT	PFS, OS
N. Matsubara	2023	Japan	NCT03474107	64 (74.4%)	22 (25.6%)	0-1	36	70.0 (58-81)	Enfortumab vedotin 1.25 mg/kg, IV, d1, d8, d5, q28d	50	66 (44-87)	TPC (paclitaxel 175 mg/m2, docetaxel 75 mg/m2, or vinflunine 320mg/m2), IV, d1, q21d	≥1 systemic therapies/platinum‐based treatment	la/mUC	IV	/	10.71	RCT	OS, PFS, ORR, AES
P. H. O’Donnell	2023	America	NCT03288545	110 (73.8%)	39 (26.2%)	0-2	76	71.0 (51-79)	Enfortumab vedotin 1.25 mg/kg, IV, d1, d8, d5, q28d	73	74 (56-89)	Enfortumab vedotin 1.25 mg/kg, IV, d1, d8,d5,q28d+pembrolizumab 200 mg d1, q21d	Previously untreated	la/mUC	IV	/	14.8	RCT	ORR, DCR, AES
T. Powles	2021	British	NCT03474107	470 (77.3%)	138 (22.7%)	0-1	301	68.0 (34–85)	Enfortumab vedotin 1.25 mg/kg, IV, d1, d8, d5, q28d	307	68 (30–88)	TPC (paclitaxel 175 mg/m2, docetaxel 75 mg/m2, or vinflunine 320mg/m2), IV, d1, q21d	Piror platinum chemotherapy	aUC	IV	/	11.1	RCT	OS, PFS, ORR, DCR, AES
X. Yan	2025	China	NCT04073602	13 (68.4%)	6 (31.6%)	0-1	19	64.0 (36, 77)	Disitamab vedotin 2mg/kg, IV, q2w	/	/	/	≥2 lines of previous systemic treatment, prior ICI therapy.	mUC	IV	HER2-	31.1	Single	ORR, OS, PFS, DCR, AES
X. Yan	2025	China	NCT04073602	8 (61.5%)	5 (38.5%)	0-1	13	64.0 (36, 77)	Disitamab vedotin 2mg/kg, IV, q2w	/	/	/	≥2 lines of previous systemic treatment, prior ICI therapy.	mUC	IV	HER2-	/	Single	ORR, OS, PFS, DCR, AES
X. Yan	2025	China	NCT04073602	5 (83.3%)	1 (16.7%)	0-1	6	63.0 (37, 73)	Disitamab vedotin 2mg/kg, IV, q2w	/	/	/	≥2 lines of previous systemic treatment, prior ICI therapy.	mUC	IV	HER2-	/	Single	ORR, OS, PFS, DCR, AES
D. Wang	2025	China	/	17 (63%)	10 (37%)	0-2	27	64.0 (39–76)	Disitamab vedotin 2mg/kg, IV, q2w	/	/	/	Treatment with RC48-ADC either as monotherapy or in combination with ICI	la/mUC	IV	HER2-	13.77	RWS	ORR, AES
W. Qu	2024	China	NCT04839510	28 (65.1%)	15 (34.9%)	0-1	43	60.0 (34-73)	Trastuzumab vedotin 2.2mg/kg, IV, q3w	/	/	/	Progressed ≥1line systemic chemotherapy	la/mUC	IV	HER2+	9.7	Single	ORR, OS, PFS, DCR, AES
B. A. McGregor	2023	America	NCT04724018	18 (78.0%)	6 (22.0%)	0-1	24	70.0 (41-88)	Sacituzumab govitecan 8 mg/kg+Enfortumab vedotin 1.25 mg/kg IV, d1, d8, q21d	/	/	/	≥1 platinum therapy/mmunotherapy/chemotherapy	mUC	IV	/	14	Single	ORR, DCR, AES
Elisabeth G. E.	2023	Netherlands	NCT02999672	12 (92.3%)	1 (7.7%)	0-1	13	62.0 (32– 78)	Trastuzumab emtansine 2.4mg/kg once weekly (qw) or 3.6mg/kg, q3w	/	/	/	≥1 standard therapeutic regimen	aUC	IV	HER2-	7.39 (4.11-10.02)	Single	ORR, OS, PFS, DCR, AES
Evan Y Yu	2021	America	NCT03219333	66 (74.0%)	23 (26.0%)	0-2	89	75.0 (68–78)	Enfortumab vedotin 1.25 mg/kg, IV, d1, d8, d5, q28d	/	/	/	Naïve or had progressed after systemic chemotherapy	mUC	IV	/	13.4 (11.3–18.9)	Single	ORR, OS, PFS, DCR, AES
Xinan Sheng	2021	China	NCT03507166	33 (76.7%)	10 (23.3%)	0-1	43	64.0 (45-75)	Disitamab vedotin 2mg/kg, IV, q2w	/	/	/	Progressed on ≥1 line of systemic chemotherapy	la/mUC	IV	HER2+	20.3 (19.9–21.6)	Single	ORR, OS, PFS, DCR, AES
A. Bardia	2021	America	NCT01631552	/	/	0-1	45	61.0 (31-90)	Sacituzumab govitecan 10 mg/kg IV on d1, d8, q21d	/	/	/	≥1 prior standard therapeutic regimen	mUC	IV	/	8.97 (0.26-55.72)	Single	ORR, OS, PFS, DCR, AES
Rosenberg	2020	America	NCT02091999	111 (72.0%)	44 (28.0%)	0-1	155	67.0 (24-86)	Enfortumab vedotin 1.25 mg/kg IV on d1, d8, d5, q28d	/	/	/	Previously treated with anti–PD-(L)1 therapy	mUC	IV	/	16.4	Single	ORR, OS, PFS, DCR, AES
J.E. Rosenberg	2019	America	NCT03219333	88 (70.0%)	37 (30.0%)	0-1	125	69.0 (40-84)	Enfortumab vedotin 1.25 mg/kg, IV, d1, d8, d5, q28d	/	/	/	Failed ≥1 line of systemic therapy	la/mUC	IV	/	10.2 (0.5-16.5)	Single	ORR, OS, PFS, DCR, AES
Erika Hamilton	2024	America	NCT03523572	27 (90.0%)	3 (10.0%)	0-1	30	72.3 (41.4–80.5)	Trastuzumab deruxtecan 5.4 mg/kg+nivolumab 360 mg, q3w	/	/	/	≥1 prior standard therapeutic regimen (expect antibody or drug specifically targeting T-cell costimulation or checkpoint pathways)	mUC	IV	HER2+	9.2 (0.3–21.3)	Single	ORR, OS, PFS, DCR, AES
S. Li	2024	China	NCT04995419	31 (77.5%)	9 (22.5%)	0-1	40	62.0 (41-75)	Enfortumab vedotin 1.25 mg/kg IV on d1, d8, d5, q28d	/	/	/	Previously treated with platinum/anti–PD-1/L1 therapy	la/mUC	IV	HER2-	6.5	Single	ORR, OS, PFS, DCR, AES
S. T. Tagawa	2021	America	NCT03547973	88 (78.0%)	25 (22.0%)	0-1	113	66.0 (33-90)	Sacituzumab govitecan 10 mg/kg IV on d1, d8, q21d	/	/	/	Recurred within 12 months after plati-num therapy	la/mUC	IV	/	9.1 (0-19.9)	Single	ORR, OS, PFS, DCR, AES
S. Takahashi	2020	Japan	NCT03070990	8 (88.9%)	1 (11.1%)	0-1	9	67.0 (61, 82)	Enfortumab vedotin 1.0 mg/kg d1, d8, d15, q28d	/	/	/	Failed ≥1 line of systemic therapy	la/mUC	IV	/	/	Single	ORR, DCR, AES
S. Takahashi	2020	Japan	NCT03070990	7 (87.5%)	1 (12.5%)	0-1	8	67.5 (57, 78)	Enfortumab vedotin 1.25 mg/kg, IV, d1, d8, d5, q28d	/	/	/	Failed ≥1 line of systemic therapy	la/mUC	IV	/	/	Single	ORR, DCR, AES
S. Takahashi	2020	Japan	NCT03070990	15 (88.2%)	2 (11.8%)	0-1	17	67.0 (57, 82)	Enfortumab vedotin 1.0 mg/kgIV Days 1, 8, 15 q28d	/	/	/	Failed ≥1 line of systemic therapy	la/mUC	IV	/		Single	ORR, PFS, DCR, AES
W. Wahafu	2024	China	NCT06178601	8 (61.5%)	5 (38.5%)	0-1	13	72.0 (61-83)	Enfortumab vedotin 1.25/1.0 mg/kg d1, d8, d15, q28d	/	/	/	/	la/mUC	IV	/	/	Single	ORR, DCR, AES
J. Zhang	2025	China	NCT05216965	35 (68.6%)	16 (31.4%)	0-1	51	68.0 (41-78)	Bulumtatug Fuvedotin 1.25 mg/kg, d1, d8, d15, q28d	/	/	/	Failed ≥1 line of systemic therapy	la/mUC	IV	/	8.6	Single	ORR, OS, PFS, DCR, AES
T. Zhang	2025	China	/	21 (77.8%)	6 (22.2%)	0-1	27	67.2 (45–84)	Disitamab vedotin 2 mg/kg+PD-1 Inhibitor, IV, q2w	/	/	/	No prior treatment or completion of postoperative treatment more than six months prior	la/mUC	IV	HER2+	/	RWS	ORR, PFS, DCR, AES
L. Zhou	2025	China	NCT04264936	22 (53.7%)	19 (46.3%)	0-1	41	66.0 (42.0-76.0)	Disitamab vedotin 1.5/2.0 mg/kg, IV+toripalimab 3.0 mg/kg, IV, q2w	/	/	/	Naïve or had progressed after systemic chemotherapy	la/mUC	IV	HER2+	33.2 (29.0-37.8)	Single	ORR, OS, PFS, DCR, AES
Capucine Baldini	2023	France	NCT04561362	14 (59.0%)	10 (41.0%)	0-1	24	66.0	Zelenectide pevedotin 5 mg/m2 every week, IV, q28d	/	/	/	≥1 prior standard therapeutic regimen	la/mUC	IV	/	11	Single	ORR, DCR
Shusuan Jiang	2024	China	NCT06079112	/	/	0-1	40	66.5 (36-78)	Bulumtatug Fuvedotin 1.25mg/kg, d1, d8+Toripalimab 240mg, IV, d1, q21d	/	/	/	/	la/mUC	IV	/	/	Single	ORR, DCR, AES
Patrizia Giannatempo	2025	British	NCT04561362.	/	/	0-2	22	77.0	Zelenectide pevedotin 5 mg/m2 on d1, d8, d15+pembrolizumab 200 mg d1, q21d	/	/	/	/	la/mUC	IV	/	7.1 (1.0–13.2)	Single	ORR, DCR
R. K. Jain,	2025	America	NCT04963153	/	/	0-1	9	/	Enfortumab vedotin 1.25 mg/kg, IV, d1, d8, d5, q28d	/	/	/	Progressed after platinum and/or PD1/L1 inhibitor therapies	mUC	IV	HER2 status	/	Single	ORR, OS, PFS, DCR, AES
Christopher J. H	2022	America	NCT04223856	36 (80.0%)	9 (20.0%)	0-2	45	69.0 (51-90)	Enfortumab vedotin 1.25 mg/kg, IV, d1, d8, d5, q28d	/	/	/	Previous adjuvant or neoadjuvant platinum-based therapy was not permitted within 12 months of the study	la/mUC	IV	/	/	Single	ORR, AES
D. Ye	2025	China	NCT04152499	/	/	0-1	11	62.0	Sacituzumab govitecan 10 mg/kg IV, d1, d8, q21d	/	/	/	Progressed on ≥1 line of systemic chemotherapy	aUC	IV	/	9.5 (7.5–16.2)	Single	ORR, PFS, DCR, AES
D. Ye	2025	China	NCT04152499	/	/	0-1	38	61.0	Sacituzumab govitecan 10 mg/kg IV, d1, d8, q21d	/	/	/	Progressed on ≥1 line of systemic chemotherapy	aUC	IV	/	11.7 (7.8–17.4)	Single	ORR, OS, PFS, DCR, AES
Ji-Ming Yao	2025	China	/	39 (76.5%)	12 (23.5%)	0-1	51	67.0 (39-86)	Disitamab Vedotin 2.0 mg/Kg,q2w+toripalimab3.0 mg/Kg/tislelizumab 200 mg, IV, q3w	/	/	/	Prior platinum-based chemotherapy	la/mUC	IV	/	20.3	Single	ORR, PFS, DCR, AES

RCT, randomized controlled trial; la/mUC, locally advanced or metastatic UC; HER2+/-, human epidermal growth factor receptor 2- positive/negative; ORR, objective response rate; OS, overall survival; PFS, progression-free survival; DCR, disease control rate; AEs, adverse events.

### 3.3 Quality assessment

Overall, the quality assessment of the 6 RCTs and 25 single-arm studies was adequately reported. Among the 5 RCTs, the overall risk of bias was assessed as low (**Supplementary Figure 1**, **Supplementary Table 2**), with 1 study raising some concerns. Four included RCTs were open-label and were rated as having “some concerns” in the randomization process. A detailed risk-of-bias assessment for each RCT is provided in **Supplementary Figure 2**, **Supplementary Table 3**. For the single-arm studies, 4 trials were classified as low risk, 16 as moderate risk, and 5 as high risk. The most frequent source of bias was confounding, primarily due to inadequate adjustment for baseline characteristics or prognostic factors. Biases related to selection and outcome measurement were also common, particularly in studies lacking blinding or standardized outcome definitions. The overall results of the GRADE assessment are summarized in **Supplementary Table 4**. The certainty of evidence for these outcomes ranged from very low to high. Downgrading was primarily due to imprecision and indirectness. For some outcomes, the evidence was rated as low or very low due to the absence of direct comparative data.

### 3.4 NMA results of efficacy and safety

#### 3.4.1 Overall survival

**Figure 2:**
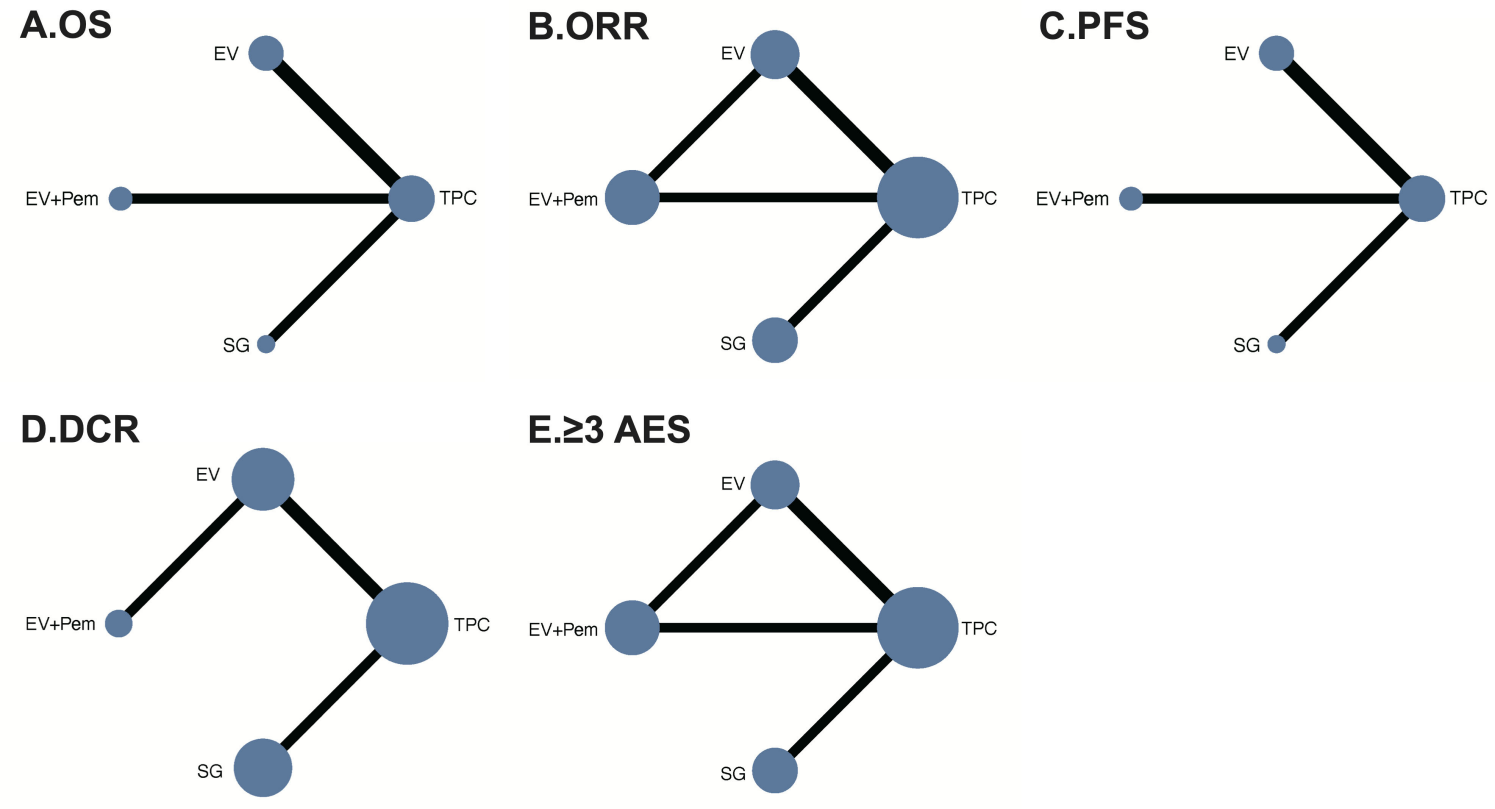
Network plot of interventions included in the network meta-analysis. **(A)** OS (overall survival); **(B)** ORR (objective response rate); **(C)** PFS (progression free survival); **(D)** DCR (disease control rate); **(E)** ≥3 AES (adverse events). Each circular node represents a type of intervention. Circle size is proportional to the total number of patients. Connecting lines indicate direct comparisons of interventions, and their width is proportional to the number of pairwise comparisons. TPC, treatment of physician’s choice; SG, sacituzumab govitecan; EV, enfortumab vedotin; Pem, pembrolizumab.

**Figure 3:**
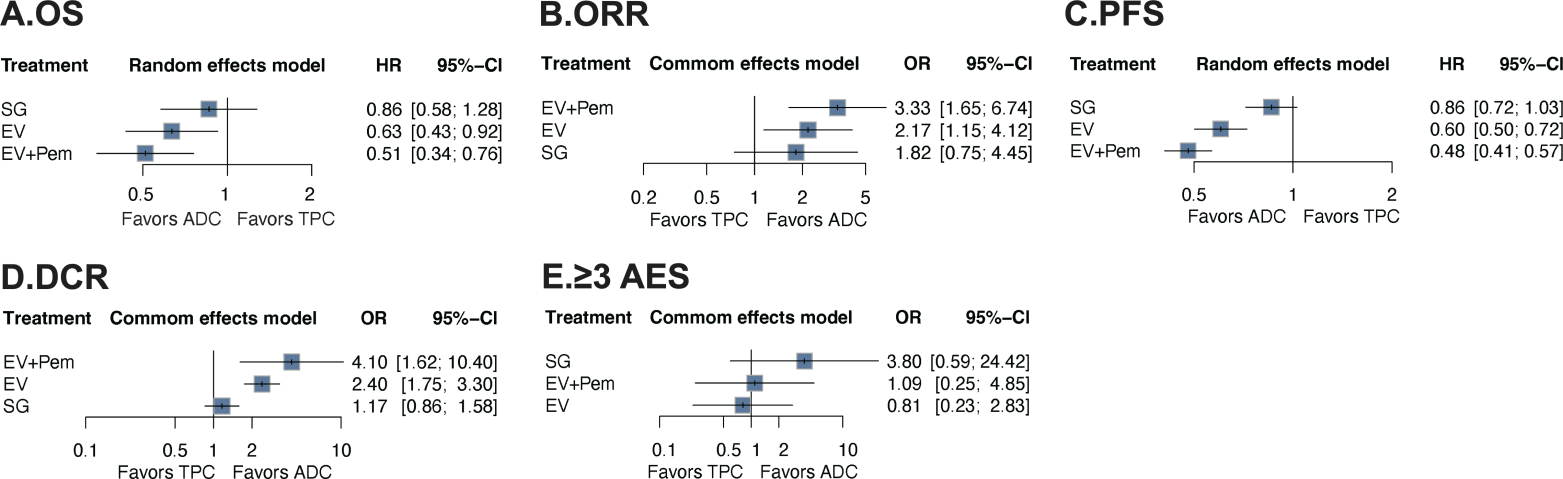
Forest plot for randomized controlled trials. **(A)** OS (overall survival); **(B)** ORR (objective response rate); **(C)** PFS (progression free survival); **(D)** DCR (disease control rate); **(E)** ≥3 AES (adverse events rate). TPC, treatment of physician’s choice; SG, sacituzumab govitecan; EV, enfortumab vedotin; Pem, pembrolizumab; ADC, antibody-drug conjugates; HR, hazard ratio; OR, odds ratio; CI, confidence intervals.

**Figure 4:**
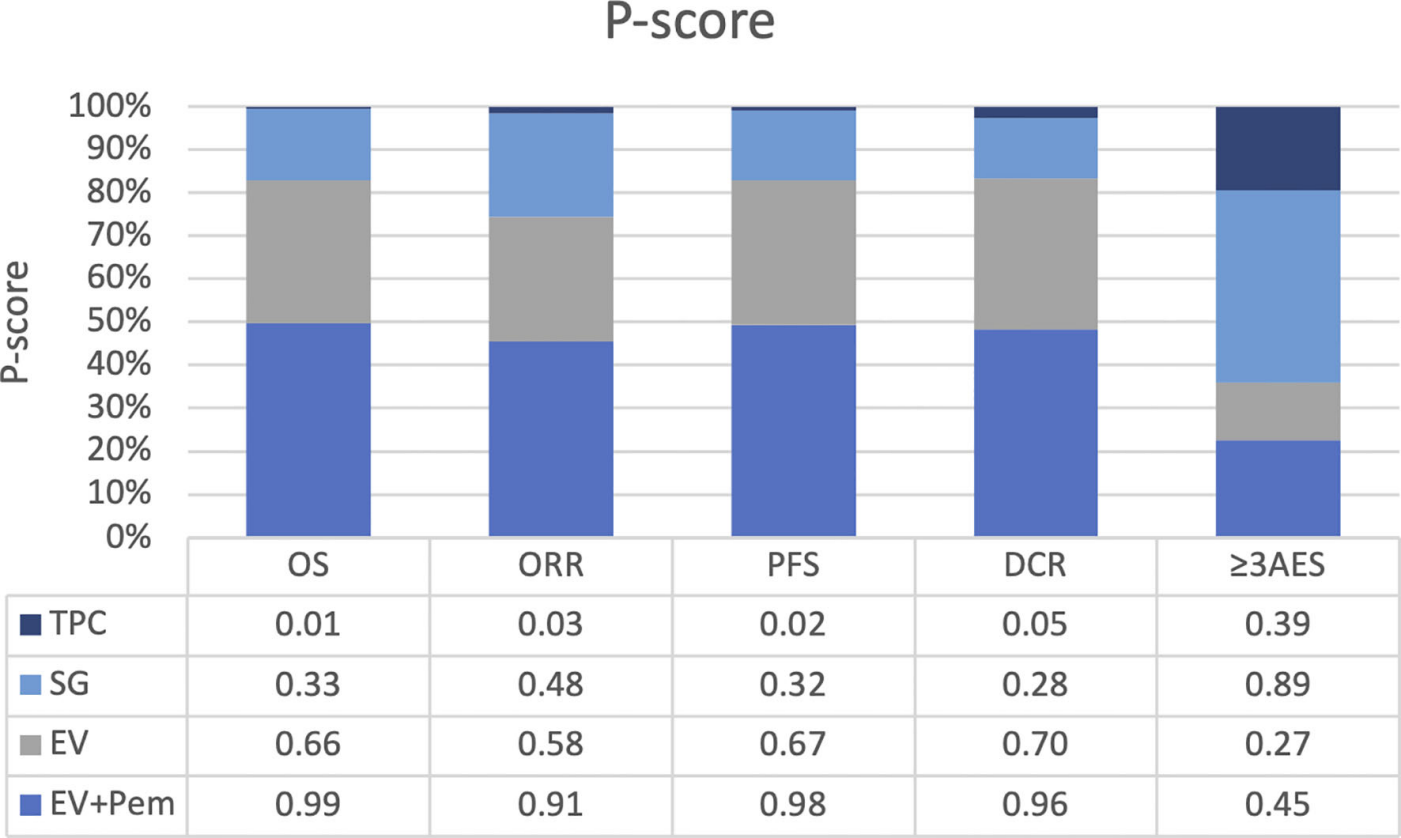
Interventions based on *P*-values for each outcome. OS, overall survival; ORR, objective response rate; PFS, progression free survival; DCR, disease control rate; AES, adverse events; TPC, treatment of physician’s choice; SG, sacituzumab govitecan; EV, enfortumab vedotin; Pem, pembrolizumab.

The analysis of OS included 4 RCTs (n=2,291). **Figure 2A** showed the network plot. As shown in **Figures 3A**, all ADC-based regimens demonstrated improved OS. Both EV monotherapy (HR: 0.63, 95% CI: 0.43-0.92) and EV combined with pembrolizumab (HR: 0.51, 95% CI: 0.34-0.76) showed statistically significant OS benefits. The analysis exhibited mild heterogeneity (I²=29.9%). Treatment ranking based on P-scores ranked EV plus pembrolizumab first (0.99), followed by EV monotherapy (0.66), and SG monotherapy (0.33) (**Figure 4**). Detailed results of pairwise comparisons are provided in **Table 2A**.

**Table 2:** Pairwise comparisons results of network-meta analysis.

A. Overall survival (OS)			
**EV**			
1.24 (0.72, 2.15)	**EV+Pem**		
0.74 (0.43, 1.27)	0.59 (0.34, 1.04)	**SG**	
**0.63 (0.43, 0.92)**	**0.51 (0.34, 0.76)**	0.86 (0.58, 1.28)	**TPC**
B. Overall response rate (ORR)			
**EV**			
0.65 (0.30, 1.40)	**EV+Pem**		
1.19 (0.40, 3.57)	1.83 (0.59, 5.70)	**SG**	
**2.17 (1.15, 4.12)**	**3.33 (1.65, 6.74)**	1.82 (0.75, 4.45)	**TPC**
C. Progression free survival (PFS)			
**EV**			
1.26 (0.98, 1.61)	**EV+Pem**		
**0.70 (0.54, 0.91)**	**0.56 (0.44, 0.71)**	**SG**	
**0.60 (0.50, 0.72)**	**0.48 (0.41, 0.57)**	0.86 (0.72, 1.03)	**TPC**
D. Disease control rate (DCR)			
**EV**			
0.59 (0.24, 1.40)	**EV+Pem**		
**2.06 (1.33, 3.20)**	**3.52 (1.32, 9.37)**	**SG**	
**2.40 (1.75, 3.30)**	**4.10 (1.62, 10.40)**	1.17 (0.86, 1.58)	**TPC**
E. Grade ≥3 adverse events (AES)			
**EV**			
0.74 (0.16, 3.50)	**EV+Pem**		
0.21 (0.02, 2.00)	0.29 (0.03, 3.11)	**SG**	
0.81 (0.23, 2.83)	1.09 (0.25, 4.85)	3.80 (0.59, 24.42)	**TPC**

Comparisons between treatments defined in the columns versus treatments defined in the rows. Statistically significant results are highlighted in bold. Results for survival outcomes, OS and PFS, were presented as hazard ratios (HR) with 95% confidence intervals (CI). Results for dichotomous outcomes, ORR, DCR, AEs, were presented as odds ratios (OR) with 95% CIs. TPC, treatment of physician’s choice; SG, sacituzumab govitecan; EV, enfortumab vedotin; Pem, pembrolizumab. OS, overall survival; ORR, objective response rate; PFS, progression free survival; DCR, disease control rate; AES, adverse events.

#### 3.4.2 Objective response rate

All 5 RCTs reported ORR as the primary outcome. The network diagram is presented in **Figure 2B**. NMA results indicated that all ADC-based regimens showed superior efficacy compared with TPC (**Figure 3A**). EV plus pembrolizumab also demonstrated statistically significant improvements (OR: 3.33, 95% CI: 1.65-6.74). Moderate heterogeneity was observed in this analysis (I²=66.7%). The node splitting analysis shows that there is no significant inconsistency between direct comparison and indirect comparison (**Supplementary Figure 3A**). Treatment ranking analysis based on P score indicated that EV plus pembrolizumab first (0.91), followed by EV monotherapy (0.67), and SG monotherapy (0.48) (**Figure 4**). The results of pairwise comparisons were showed in **Table 2B**.

#### 3.4.3 Progression free survival

Four RCTs were included in the analysis of PFS. The network diagram was shown in **Figure 2C**. All ADC-based regimens were associated with PFS improvement compared with TPC. Statistically significant benefits were observed for EV plus pembrolizumab (HR: 0.48, 95% CI: 0.41-0.57), and EV monotherapy (HR: 0.60, 95% CI: 0.50-0.72). No substantial heterogeneity was observed (I²=0%). Treatment ranking identified EV plus pembrolizumab as the most effective regimen (0.98), followed by EV monotherapy (0.67), and SG monotherapy (0.32) (**Figure 4**). Results of all pairwise comparisons are summarized in **Table 2C**.

#### 3.4.4 Disease control rate

A total of 1,554 patients from 4 RCTs were included. The network diagram of treatment comparisons is shown in **Figure 2D**. All ADC-based regimens were associated with higher DCR (**Figure 2D**). Statistically significant improvements were observed for EV plus pembrolizumab (OR: 4.10, 95% CI: 1.62-10.40), and EV monotherapy (OR: 2.40, 95% CI: 1.75-3.30). In contrast, SG monotherapy did not show a significant benefit (OR: 1.17, 95% CI: 0.86-1.58). No heterogeneity was observed in NMA (I²=0%). Treatment ranking identified EV plus pembrolizumab as the highest-ranked regimen (0.96), followed by EV monotherapy (0.70), and SG monotherapy (0.28) (**Figure 4**). Results of all pairwise comparisons are presented in **Table 2D**.

#### 3.4.5 Safety (Grade ≥3)

All included RCTs reported the incidence of grade ≥3 AEs. The network diagram is shown in **Figure 2E**. Compared with TPC, ADC-based treatments did not demonstrate a statistically significant in grade ≥3 AEs. EV monotherapy showed a non-significant trend toward lower AE risk (OR: 0.81, 95% CI: 0.23-2.83; P-score: 0.27) (**Figure 3E**). Node-splitting analysis indicated no significant inconsistency between direct and indirect evidence (**Supplementary Figure 3B**). High heterogeneity was present in the analysis (I²=86.4%). Treatment ranking identified SG monotherapy was the least favorable option (0.89), follower by EV plus pembrolizumab (0.45) (**Figure 4**). **Table 2E** showed the results of all pairwise comparisons.

### 3.5 Meta-analysis of single trials

Single-arm trials can provide a critical source of evidence for new therapeutic agents, especially when randomized data are not yet mature or available (53). To complement our primary RCT-based network meta-analysis, we performed a meta-analysis of single-arm studies to describe the aggregated efficacy and safety characteristics of various ADC regimens.

#### 3.5.1 Median overall survival

The pooled mOS across all regimens was 12.96 months (95% CI: 10.38-16.17; **Supplementary Figure 4A**). The combination of DV and TP showed the longest mOS, at 33.1 months (95% CI: 22.41-48.89), followed by TV monotherapy with an mOS of 14.90 months (95% CI: 11.76-18.88). Although the funnel plot was approximately symmetrical, Egger’s test suggested the presence of publication bias (*P* = 0.032; **Supplementary Figure 5A**).

#### 3.5.2 Objective response rate

The meta-analysis of 25 single-arm studies demonstrated pooled ORR was 49% (95% CI: 42%-56%, **Supplementary Figure 4B**). Among combination therapies, BF plus toripalimab (TP) yielded the highest pooled ORR of 88% (95% CI: 73%-96%). Among monotherapies, EV had a pooled ORR of 51% (95% CI: 37%-66%), DV monotherapy showed a pooled ORR of 47% (95% CI: 43%-52%). However, SG monotherapy had a comparatively lower pooled ORR of 29% (95% CI: 23%-35%). The funnel plot was is basically symmetrical on both sides, and Egger’s test (*P* = 0.987) indicated no significant publication bias (**Supplementary Figure 5B**).

#### 3.5.3 Mediam progression free survival

The overall mPFS was 6.29 months (95% CI: 5.66-6.98; I²=79.9%, P<0.001; **Supplementary Figure 4C**). The combination of DV and TP achieved the longest median PFS as 9.30 months (95% CI: 6.55-13.04), followed by DV combined with ICI at 7.42 months (95% CI: 5.63-9.31). Among ADC monotherapies, EV at 1.0 mg/kg showed the best outcome, with a mPFS of 8.10 months (95% CI: 4.97-13.20). The funnel plot and Egger’s test (*P* = 0.316) indicated No significant publication bias (**Supplementary Figure 5C**).

#### 3.5.4 Disease control rate

The overall pooled DCR across regimens was 79% (95% CI: 70%-85%), with moderate heterogeneity among studies (I²=72.5%, P<0.0001; **Supplementary Figure 4D**). BF plus TP showed the highest pooled DCR of 93% (95% CI: 80%-98%), followed by DV plus TP (88%; 95% CI: 74%-96%) and DV combined with ICI (87%; 95% CI: 66%-97%). Among monotherapies, DV showed the highest pooled DCR (93%; 95% CI: 84%-97%), followed by TV (84%; 95% CI: 69%-98%), while T-DM1 had a relatively low DCR of 38% (95% CI: 14%-68%). The funnel plot and Egger’s test *(P* = 0.147) indicated no significant publication bias (**Supplementary Figure 5D**).

#### 3.5.5 Safety (Grade ≥3)

Data from single-arm studies showed that the overall pooled incidence of grade ≥3 AEs was 52% (95% CI: 35%-68%), with high heterogeneity among studies (I²=82.7%, P<0.001; **Supplementary Figure 4E**). Among combination therapies, SG plus EV was associated with the highest incidence (78%; 95% CI: 56%-93%), while BF plus toripalimab had the lowest (23%; 95% CI: 11%-38%). Among monotherapies, TV showed the highest incidence (100%; 95% CI: 92%-100%), followed by SG monotherapy (78%; 95% CI: 27%-97%). The funnel plot and Egger’s test (*P* = 0.050) indicated no significant publication bias (**Supplementary Figure 5E**).

### 3.6 Meta regression and subgroup analysis

Network meta-regression analysis indicated that covariates including age, prior therapy, and follow-up duration showed no statistically significant association with efficacy outcomes (OS, ORR, PFS, DCR) or the safety outcome (AES), as all 95%CIs included zero. This suggests that observed heterogeneity in treatment effects may be attributable to other unmeasured factors or random variation. Detailed regression coefficients and intervals are provided in **Supplementary Table 5**. For single-arm trials, regression results further confirmed prior treatment with ≥2 lines (*P* = 0.021) and Chinese origin (*P* = 0.003) were significantly correlated with DCR. Patients from the Netherlands had significantly shorter PFS (coeff=-0.97, *P* = 0.009), and treatment for ≥12 months was associated with significantly fewer AES (coeff=-2.02, *P* = 0.004). No other covariates showed statistically significant associations in regression models (**Supplementary Table 6**).

Subgroup analyses of single-arm studies identified several significant influencing factors (**Supplementary Table 7**). Tumor molecular subtype exhibited a substantial impact on both mPFS and mOS (*P* < 0.001). Treatment duration was also a key prognostic factor, with patients treated for ≥12 months demonstrating significantly improved ORR (*P* < 0.001) and a lower incidence of AES (*P* = 0.030) compared to those treated for a shorter duration. Country-specific variations were also significant, with Chinese patients achieving superior DCR (*P* < 0.001) and the longest mPFS (*P* = 0.002). Furthermore, the number of prior lines of therapy significantly influenced DCR (*P* = 0.001).

### 3.7 Sensitive analysis

Two sensitivity analyses were performed to verify the robustness of the NMA results. First, after excluding small-sample studies, heterogeneity significantly decreased across all outcome indicators (I² =0% in all cases), particularly for ORR and AEs, suggesting that small-sample studies were a major source of heterogeneity. The primary conclusions remained unchanged, supporting the stability of the results. Second, after excluding studies in which the control group not received TPC, the analysis results remained robust. In summary, the sensitivity analyses further confirm the reliability of the main study findings (**Supplementary Table 8**, **Supplementary Figure 6**). Additionally, a leave−one−out sensitivity analysis of the single−arm study results confirmed the consistency of the findings. These sensitivity analyses further affirm the reliability of the main study conclusions (**Supplementary Figure 7**).

## 4 Discussion

ADCs represent an effective therapeutic strategy for a variety of cancers (54). By combining the potent cytotoxicity of chemotherapeutic agents with the precise targeting capability of monoclonal antibodies, ADCs selectively bind to tumor cells and deliver highly cytotoxic payloads directly to the tumor site (55, 56). Owing to the high specificity and affinity of antibodies for particular epitopes on target antigens, ADCs minimize off-target effects, enhance drug delivery efficiency, and improve the therapeutic index (57). Currently, more than 100 ADCs are under clinical investigation (58). Multiple meta-analyses have demonstrated that ADCs exhibit favorable efficacy in patients with la/mUC, the combination of EV and pembrolizumab represents the most effective first-line treatment for UC, while EV monotherapy shows the highest efficacy among single-agent regimens. Additionally, the combination of SG and EV also yield superior outcomes in advanced UC (9–11). Nevertheless, novel ADCs continue to emerge, such as MRG002, ZP, BF, T-DXd, and T-DM1. In light of ongoing clinical trial advancements and emerging ADC agents, this study systematically incorporates 30 clinical trials (5 RCTs and 25 single-arm studies) to provide the most comprehensive evaluation to date of the efficacy and safety of ADC therapies, both as monotherapy and in combination regimens, for la/mUC. Through detailed subgroup analyses, this meta-analysis not only confirms the efficacy of ADCs, but also highlights key considerations for optimizing patient stratification, treatment sequencing, and the design of future clinical trials.

NMA results demonstrated that, compared with TPC, the combination of EV and pembrolizumab yielded significant improvements in ORR, PFS, and OS, while maintaining an acceptable safety profile. These findings are consistent with results from pivotal phase III trials such as EV-302 (26) and previous studies (11), marking the advent of a new era in urothelial carcinoma treatment characterized by targeted-immune combination therapy. EV is a fully humanized monoclonal antibody (AGS-22M6) directed against nectin-4 and conjugated to the microtubule-disrupting agent monomethyl auristatin E (MMAE) (59). Nectin-4 is frequently overexpressed in urothelial malignancies and has been associated with disease progression and poor prognosis (60). Upon binding to nectin-4, EV is internalized into cells, where MMAE is released, leading to disruption of the microtubule network, cell cycle arrest, and ultimately apoptosis (61). The observed efficacy of EV in combination with pembrolizumab may be attributed to its ability to modulate the tumor microenvironment and enhance the activity of immune checkpoint inhibitors, including dendritic cell activation and induction of immunogenic cell death, that may amplify the overall antitumor immune response (62–64). The results of the NMA suggest that EV may offer safety advantages. Study have confirmed that EV extends survival while maintaining patient quality of life (65). This high selectivity toward nectin-4-expressing tumor cells likely forms an important basis for EV’s potential safety benefits. Meanwhile, in combination with the results of the single-arm study, we found that for EV, the 1.25 mg/kg dose had a better efficacy in improving the ORR than the 1.0 mg/kg group. More research is still needed in the future to determine the optimal dose. In the future, further research is warranted to validate these findings. SG is a Trop-2-directed antibody-drug conjugate that delivers the cytotoxic payload SN-38 directly into tumor cells expressing high Trop-2 levels (66). However, SG did not show significant superiority over TPC across most efficacy endpoints, which may be attributable to its use in later-line settings and its distinct mechanism of action as a topoisomerase I inhibitor-based agent (67). For safety outcome, SG were associated with a higher incidence of grade ≥3 AEs, which may attributable to bone marrow suppression and gastrointestinal toxicity induced by the SN-38 payload (68, 69). These toxicities warrant particularly close monitoring in heavily pretreated patients. Despite this, the overall treatment discontinuation rate due to toxicity was only 15%, indicating that most patients can tolerate the regimen with appropriate supportive care (27). This presents a key trade-off in clinical decision-making: SG remains an important option for patients with good performance status who can be closely monitored and have their toxicities managed promptly; however, greater caution is warranted for those who are frail or have significant comorbidities.

Among the novel therapeutic regimens evaluated, BF combined with TP demonstrated the most robust efficacy in terms of response rates, achieving a pooled ORR of 88% and a DCR of 93%. Both BF (9MW2821) and EV share a core mechanism of action, targeting Nectin-4 and delivering the cytotoxic payload MMAE (70). However, BF differs in its utilization of site-specific conjugation technology and a novel linker, IDconnect. This optimization resulted in significantly higher intratumoral MMAE exposure for BF compared to EV in preclinical studies, translating to superior antitumor activity (71). In a first-in-human, phase I/II study conducted by J Zhang et al. (49), BF demonstrated a manageable safety profile and clinically meaningful efficacy. TP is a monoclonal anti-PD-1 antibody that specifically binds to PD-1, thereby reversing the immune suppression of T cells by tumor cells and enhancing T-cell-mediated recognition and killing (72). Compared to Pembrolizumab, TP induced a more robust inflammatory response in immune cells *in vitro* (73). Consequently, the effectiveness of the BF and TP combination may stem from the synergy between “targeted cytotoxicity” and “immune activation.” Concurrently, our analysis revealed promising efficacy for TV, T-DM1, and T-DM1 in combination with nivolumab. TV targets HER2 and delivers the MMAE (74). Its observed OS benefit may stem from the potential of the MMAE payload to induce immunogenic cell death. Similarly, T-DXd targets HER2 but delivers a topoisomerase I inhibitor (DXd) (75). When combined with nivolumab, the ability of T-DXd to remodel the tumor microenvironment may synergize with immunotherapy, leading to the observed broad efficacy across multiple endpoints (32). T-DM1 combines the HER2-targeting properties of trastuzumab with the cytotoxic activity of the microtubule inhibitor DM1 (also known as mertansine or emtansine) (76). Although extensive studies have established the efficacy and generally manageable safety profile of T-DM1 in breast cancer, particularly in HER2-positive patients (77–79), its response rate in UC appears limited. However, this conclusion is tempered by the availability of only a single relevant study, which was further limited by a small sample size. That study also highlighted challenges such as a low HER2-positivity rate and difficulties in patient recruitment, which hindered further investigation (30). Consequently, the therapeutic potential of T-DM1 in UC requires validation in larger, dedicated studies to adequately assess its efficacy and safety profile. However, due to the limited number of included articles and the inherent limitations of the design of the single-arm studies, these results need to be interpreted with caution.

HER2 overexpression is a well-established poor prognostic indicator in UC, typically associated with more aggressive disease, higher risk of metastasis, and shorter OS (80). DV is an ADC drug which target HER2, which comprises a cleavable valine-citrulline linker conjugated to the microtubule inhibitor MMAE (81). DV is currently approved in China for the treatment of urothelial carcinoma and has been granted permission to conduct Phase II clinical trials directly in the U.S (82). It specifically binds to HER2, induces internalization, and thereby inhibits tumor cell proliferation (83, 84). Substantial evidence has confirmed the efficacy of DV in HER2-positive urothelial cancer, with its therapeutic potential likely increasing alongside higher HER2 expression levels (85). Notably, the study by X. Yan (45) has expanded the potential application of DV to patients with HER2-low expression, demonstrating significant antitumor activity and a manageable safety profile even in heavily pretreated metastatic UC. Our integrated analysis of two studies (45, 46) focusing on the HER2-low population similarly indicated clinical benefit in this subgroup, reflected by prolonged mOS and a higher DCR. Pooled analyses and subgroup evaluations from single-arm studies further support the robust antitumor activity of DV-based regimens, whether administered as monotherapy or in combination with ICI or TP. Subgroup analyses suggested that patients with higher HER2 expression derived more pronounced benefits in terms of mPFS and ORR, while the combination of DV and TP was associated with the longest survival outcomes. Overall, DV demonstrates compelling efficacy and a manageable safety profile. As the included RCTs were not designed with predefined subgroup analyses for this factor, no further subgroup or regression analyses were performed in this study to explore its impact. Future efforts should prioritize prospective studies, particularly large-scale RCTs in HER2-low populations, coupled with comprehensive biomarker analyses, to further refine appropriate treatment strategies.

The safety results from the NMA indicated a higher incidence of grade ≥3 adverse events associated with ADC drugs, which was further supported by the analysis of single−arm studies. These toxicities are largely attributed to the cytotoxic effects of ADC payloads, which disrupt the cell cycle and elicit systemic inflammatory responses (86), underscoring the importance of proactive supportive care and prophylactic medication in the management of these regimens. Although the rate of toxicities was high, most AEs could be managed through dose adjustments, prophylactic use of granulocyte colony-stimulating factor (G-CSF), and supportive measures. Treatment discontinuation due to intolerance was infrequent (87, 88). However, cumulative toxicity should be closely monitored in the context of early-line and combination therapies, especially in patients with limited bone marrow reserve resulting from prior treatments (89–91).

Furthermore, subgroup analyses and meta-regression identified treatment duration and geographic region as significant factors associated with treatment outcomes. Patients who received therapy for ≥12 months demonstrated significantly higher ORR and DCR, along with a more favorable safety profile, providing a compelling rationale for maintaining an adequate treatment course whenever tolerated. Concurrently, the subgroup of Chinese patients exhibited superior disease control and prolonged progression-free survival. The superior outcomes observed in Chinese patients may be attributable to a combination of factors, including ethnic differences in tumor biology or pharmacogenomics, variations in clinical practice patterns (e.g., earlier and more intensive supportive care), or potentially a higher prevalence of specific molecular subtypes of UC that are more responsive to ADC therapy in the studied population (92, 93). While this observation highlights the potential influence of regional variations in tumor biology or clinical practice, its underlying drivers require validation in prospective studies. However, it is important to emphasize that these findings derive from pooled single-arm analyses, and their interpretation should remain cautious and strictly exploratory, as no significant factors were identified in the meta-regression of RCTs.

Several limitations of this study should be acknowledged. First, the number of RCTs is limited for certain treatment comparisons, particularly those involving newer therapeutic agents. Second, the quality of evidence assessed using the GRADE framework varied substantially across outcomes, ranging from very low to high, with downgrading primarily due to imprecision, indirectness, or inconsistency. This may be attributed to the absence of head-to-head comparative studies for some interventions. Third, variations in baseline patient characteristics across the included studies could introduce potential effect modification and limit the generalizability of the findings to broader populations. Although we explored potential sources of heterogeneity through sensitivity analyses and network meta-regression, the results should still be interpreted with caution. Finally, some relevant studies were available only as conference abstracts, which restricted the detailed information necessary for subgroup analyses and adjustment of potential confounders.

Despite these limitations, our meta-analysis remains valuable. First, we conducted a comprehensive evidence synthesis, systematically evaluating a range of marketed and investigational ADCs administered as monotherapy or in combination regimens. By analyzing data from both RCTs and single-arm studies, and employing a combination of pairwise and network meta-analyses, we provided a comprehensive comparison of the efficacy and safety profiles across different treatment options. Furthermore, meta-regression, sensitivity analysis and subgroup analyses were performed, offering preliminary insights into potential influencing factors and the stability of the results. These findings provide valuable implications for future individualized treatment strategies and research directions. With ongoing clinical exploration of ADC-based combination immunotherapies and the expanding application of diverse ADC types in advanced genitourinary cancers, we anticipate that more promising therapeutic options will become available for patients in the near future.

## 5 Conclusion

This meta-analysis establishes that the therapeutic future of la/mUC lies in combining ADCs with immunotherapy. The superior efficacy of EV plus pembrolizumabunderscore the potent synergy between targeted payload delivery and immune checkpoint blockade. Our integrated results provide a critical, hierarchical profile of ADC-based regimens, crucial for clinical decision-making. This includes remarkable response rates with novel combinations like BF plus TP, T-DM1 plus nivolumab, expanded potential of DV in HER2-low populations, and a necessary caution for SG-based therapies due to their distinct toxicity profile. Collectively, these findings mark a significant advance toward precision immuno-oncology in la/mUC, positioning ADCs as pivotal agents for activating and enhancing antitumor immunity.

## Data Availability

The original contributions presented in the study are included in the article/**Supplementary Material**. Further inquiries can be directed to the corresponding author.
