# Supplementary material for: Efficacy and safety of antibody-drug conjugate based therapy in locally advanced or metastatic urothelial carcinoma: a systematic review and network meta-analysis of emerging clinical evidence
Source: Front Immunol. 2026 Mar 24;17:1728521. doi: 10.3389/fimmu.2026.1728521 (PMC13053482; doi:10.3389/fimmu.2026.1728521)
Supplement: Supplementary file 1 [file Table1.docx]

**Efficacy and safety of antibody-drug conjugate based therapy in locally advanced or metastatic urothelial carcinoma: a systematic review and network meta-analysis of emerging clinical evidence**

**Supplemental material**

**Supplementary Table 1. Search strategies.**

**Supplementary Table 2. Risk of bias summary in randomized controlled trials.**

**Supplementary Table 3. Risk of bias summary in single-arm studies.**

**Supplementary Table 4. GRADE evidence assessment.**

**Supplementary Table 5. Network meta-regression results**

**Supplementary Table 6. Regression analysis for single-arm studies.**

**Supplementary Table 7. Subgroup analysis of single-arm studies.**

**Supplementary Table 8. Sensitive analysis of NMA.**

**Supplementary Figure 1. Risk of bias in randomized controlled trials.**

**Supplementary Figure 2. Risk of bias in non-randomized studies.**

**Supplementary Figure 3. Node splitting result of randomized controlled trials.**

**Supplementary Figure 4. Forest plots of single-arm trials.**

**Supplementary Figure 5. Funnel plots of single-arm trials.**

**Supplementary Figure 6. Sensitive analysis of NMA (P-score).**

**Supplementary Figure 7. Sensitive analysis of single-arm trials.**

**Supplementary Table 1. Search strategies.**

| **Pubmed** |  |
| --- | --- |
| **#1** | "transitional cell carcinoma"[MeSH] |
| **#2** | "cancer of the urothelium"[Title/Abstract] OR "carcinoma of the urothelium"[Title/Abstract] OR "carcinoma, transitional cell"[Title/Abstract] OR "metastatic transitional cell"[Title/Abstract] OR "metastatic urothelial"[Title/Abstract] OR "papillary TCC"[Title/Abstract] OR "papillary transitional cell carcinoma"[Title/Abstract] OR "papillary urothelial carcinoma"[Title/Abstract] OR "transitional cell cancer"[Title/Abstract] OR "transitional cell carcinomata"[Title/Abstract] OR "transitional cell carcinomatosis"[Title/Abstract] OR "upper tract urothelial carcinoma"[Title/Abstract] OR "urothelial cancer"[Title/Abstract] OR "urothelial carcinogenesis"[Title/Abstract] OR "urothelial carcinoma"[Title/Abstract] OR "urothelial cell cancer"[Title/Abstract] OR "urothelial cell carcinogenesis"[Title/Abstract] OR "urothelial cell carcinoma"[Title/Abstract] OR "urothelial malignancies"[Title/Abstract] OR "urothelial malignancy"[Title/Abstract] OR "urothelial metastases"[Title/Abstract] OR "urothelial metastasis"[Title/Abstract] OR "urothelium cancer"[Title/Abstract] OR "urothelium carcinogenesis"[Title/Abstract] OR "urothelium carcinoma"[Title/Abstract] |
| **#3** | "antibody drug conjugate"[MeSH] |
| **#4** | "antibody drug conjugate"[Title/Abstract] OR "drug-antibody conjugate"[Title/Abstract] OR "antibody-drug conjugate"[Title/Abstract] OR "Enfortumab Vedotin"[Title/Abstract] OR "Padcev"[Title/Abstract] OR "Sacituzumab Govitecan"[Title/Abstract] OR "Trodelvy"[Title/Abstract] OR "Trastuzumab Deruxtecan"[Title/Abstract] OR "Enhertu"[Title/Abstract] OR "Disitamab Vedotin"[Title/Abstract] OR "RC48"[Title/Abstract] OR "ASG-15ME"[Title/Abstract] OR "ASG-22ME"[Title/Abstract] OR "Telisotuzumab Vedotin"[Title/Abstract] OR "ABBV-399"[Title/Abstract] OR "Mirvetuximab Soravtansine"[Title/Abstract] OR "IMGN853"[Title/Abstract] OR "A166"[Title/Abstract] OR "ARX788"[Title/Abstract] OR "DS-8201"[Title/Abstract] OR "Zelenectide pevedotin"[Title/Abstract] OR "BT8009"[Title/Abstract] OR "9MW2821"[Title/Abstract] |
| **#5** | "clinical" OR "trial" OR "clinical trial" OR "randomized controlled trial" OR "cohort" OR "prospective" OR "retrospective" |
| **#6** | (#1 OR #2) AND (#3 OR #4) AND #5 |
| **Embase** |  |
| **#1** | 'transitional cell carcinoma'/exp |
| **#2** | ('transitional cell carcinoma' OR 'cancer of the urothelium' OR 'carcinoma of the urothelium' OR 'carcinoma, transitional cell' OR 'metastatic transitional cell' OR 'metastatic urothelial' OR 'papillary TCC' OR 'papillary transitional cell carcinoma' OR 'papillary urothelial carcinoma' OR 'transitional cell cancer' OR 'transitional cell carcinomata' OR 'transitional cell carcinomatosis' OR 'upper tract urothelial carcinoma' OR 'urothelial cancer' OR 'urothelial carcinogenesis' OR 'urothelial carcinoma' OR 'urothelial cell cancer' OR 'urothelial cell carcinogenesis' OR 'urothelial cell carcinoma' OR 'urothelial malignancies' OR 'urothelial malignancy' OR 'urothelial metastases' OR 'urothelial metastasis' OR 'urothelium cancer' OR 'urothelium carcinogenesis' OR 'urothelium carcinoma'):ti,ab,kw |
| **#3** | 'antibody drug conjugate'/exp |
| **#4** | 'antibody drug conjugate' OR 'drug-antibody conjugate' OR 'antibody-drug conjugate' OR 'Enfortumab Vedotin' OR 'Padcev' OR 'Sacituzumab Govitecan' OR 'Trodelvy' OR 'Trastuzumab Deruxtecan' OR 'Enhertu' OR 'Disitamab Vedotin' OR 'RC48' OR 'ASG-15ME' OR 'ASG-22ME' OR 'Telisotuzumab Vedotin' OR 'ABBV-399' OR 'Mirvetuximab Soravtansine' OR 'IMGN853' OR 'A166' OR 'ARX788' OR 'DS-8201' OR 'Zelenectide pevedotin' OR 'BT8009' OR '9MW2821’):ti,ab,kw |
| **#5** | ('clinical' OR 'trial' OR 'clinical trial' OR 'randomized controlled trial' OR 'cohort' OR ’prospective' OR 'retrospective'):ti,ab,kw |
| **#6** | (#1 OR #2) AND (#3 OR #4) AND #5 |
| **Cochrane Library** |  |
| **#1** | 'transitional cell carcinoma'/exp |
| **#2** | ('transitional cell carcinoma' OR 'cancer of the urothelium' OR 'carcinoma of the urothelium' OR 'carcinoma, transitional cell' OR 'metastatic transitional cell' OR 'metastatic urothelial' OR 'papillary TCC' OR 'papillary transitional cell carcinoma' OR 'papillary urothelial carcinoma' OR 'transitional cell cancer' OR 'transitional cell carcinomata' OR 'transitional cell carcinomatosis' OR 'upper tract urothelial carcinoma' OR 'urothelial cancer' OR 'urothelial carcinogenesis' OR 'urothelial carcinoma' OR 'urothelial cell cancer' OR 'urothelial cell carcinogenesis' OR 'urothelial cell carcinoma' OR 'urothelial malignancies' OR 'urothelial malignancy' OR 'urothelial metastases' OR 'urothelial metastasis' OR 'urothelium cancer' OR 'urothelium carcinogenesis' OR 'urothelium carcinoma'):ti,ab,kw |
| **#3** | 'antibody drug conjugate'/exp |
| **#4** | 'antibody drug conjugate' OR 'drug-antibody conjugate' OR 'antibody-drug conjugate' OR 'Enfortumab Vedotin' OR 'Padcev' OR 'Sacituzumab Govitecan' OR 'Trodelvy' OR 'Trastuzumab Deruxtecan' OR 'Enhertu' OR 'Disitamab Vedotin' OR 'RC48' OR 'ASG-15ME' OR 'ASG-22ME' OR 'Telisotuzumab Vedotin' OR 'ABBV-399' OR 'Mirvetuximab Soravtansine' OR 'IMGN853' OR 'A166' OR 'ARX788' OR 'DS-8201' OR 'Zelenectide pevedotin' OR 'BT8009' OR '9MW2821’):ti,ab,kw |
| **#5** | ('clinical' OR 'trial' OR 'clinical trial' OR 'randomized controlled trial' OR 'cohort' OR 'prospective' OR 'retrospective'):ti,ab,kw |
| **#6** | (#1 OR #2) AND (#3 OR #4) AND #5 |
| **Web of Science** |  |
| **#1** | TS=(transitional cell carcinoma OR cancer of the urothelium OR carcinoma of the urothelium OR carcinoma, transitional cell OR metastatic transitional cell OR metastatic urothelial OR papillary TCC OR papillary transitional cell carcinoma OR papillary urothelial carcinoma OR transitional cell cancer OR transitional cell carcinomata OR transitional cell carcinomatosis OR upper tract urothelial carcinoma OR urothelial cancer OR urothelial carcinogenesis OR urothelial carcinoma OR urothelial cell cancer OR urothelial cell carcinogenesis OR urothelial cell carcinoma OR urothelial malignancies OR urothelial malignancy OR urothelial metastases OR urothelial metastasis OR urothelium cancer OR urothelium carcinogenesis OR urothelium carcinoma) |
| **#2** | TS=(antibody drug conjugate OR drug-antibody conjugate OR antibody-drug conjugate OR Enfortumab Vedotin OR Padcev OR Sacituzumab Govitecan OR Trodelvy OR Trastuzumab Deruxtecan OR Enhertu OR Disitamab Vedotin OR RC48 OR ASG-15ME OR ASG-22ME OR Telisotuzumab Vedotin OR ABBV-399 OR Mirvetuximab Soravtansine OR IMGN853 OR A166 OR ARX788 OR DS-8201 OR Zelenectide pevedotin OR BT8009 OR 9MW2821) |
| **#3** | TS=(clinical OR trial OR clinical trial OR randomized controlled trial OR cohort OR prospective OR retrospective) |
| **#4** | #1 AND #2 AND #3 |

**Supplementary Table 2. Risk of bias summary in randomized controlled trials.**

| Study | Randomization process | Deviations from  intended interventions | Mising outcome data | Measurement of  the outcome | Selection of  the reported result | Overall Bias |
| --- | --- | --- | --- | --- | --- | --- |
| Matsubara 2023 | Some concerns | Some concerns | Low | Some concerns | Low | Some concerns |
| P. H. O'Donnell 2023 | Low | Low | Low | Low | Low | Low |
| T. Powles 2021 | Some concerns | Low | Low | Low | Low | Low |
| T. Powles 2024 | Some concerns | Low | Low | Low | Low | Low |
| T. Powles 2025 | Some concerns | Low | Low | Low | Low | Low |

**Supplementary Table 3. Risk of bias summary in single-arm trials.**

| Study | Confounding | Selection of participants | Classification  of interventions | Deviations from intended interventions | Missing data | Measurement  of outcomes | Selection of  reported results | Overall risk  of bias |
| --- | --- | --- | --- | --- | --- | --- | --- | --- |
| A.Baedia 2021 | High | Low | Low | Low | Low | Low | Low | High |
| B.A.McGregor 2024 | Moderate | Low | Low | Low | Low | Low | Moderate | Moderate |
| C.Baldini 2023 | Moderate | Low | Low | Moderate | Moderate | Low | Moderate | Moderate |
| C.J.Hoimes 2023 | Moderate | Low | Low | Moderate | Low | Low | Low | Moderate |
| D.Wang 2025 | Moderate | Low | Low | Low | Low | Low | Moderate | Moderate |
| D.Ye 2025 | High | Low | Low | Moderate | Low | Low | Low | High |
| Elisabeth G. E 2023 | Moderate | Low | Low | Low | Moderate | Low | Low | Moderate |
| Erika Hamilton 2024 | Moderate | Low | Low | Low | Moderate | Low | Low | Moderate |
| Evan Y Yu 2021 | Low | Low | Low | Low | Low | Low | Low | Low |
| Patrizia Giannatempo 2025 | Moderate | Low | Low | Low | Moderate | Low | Moderate | Moderate |
| J Zhang 2025 | Moderate | Low | Low | Low | Moderate | Low | Low | Moderate |
| J.E.Rosenberg 2019 | Moderate | Low | Low | Low | Moderate | Low | Low | Moderate |
| J.M.Yao 2025 | High | Low | Low | Low | Moderate | Moderate | Low | High |
| J.Rosenberg 2020 | Low | Low | Low | Low | Low | Low | Low | Low |
| L.Zhou 2025 | Low | Low | Low | Low | Low | Low | Low | Low |
| R.K.Jain 2025 | Moderate | Low | Low | Low | Moderate | Low | Moderate | Moderate |
| S.Li 2024 | Moderate | Low | Low | Low | Moderate | Low | Low | Moderate |
| S.T.Tagawa 2021 | Moderate | Low | Low | Low | Moderate | Low | Moderate | Moderate |
| Shusuan Jiang 2024 | Moderate | Low | Low | Low | Moderate | Low | Low | Moderate |
| T.Zhang 2025 | High | Low | Low | Moderate | Moderate | Moderate | Low | High |
| Takahashi 2020 | Moderate | Low | Low | Moderate | Moderate | Low | Low | Moderate |
| W.Qu 2024 | Low | Low | Low | Low | Moderate | Low | Low | Low |
| W.Wahafu2024 | Moderate | Low | Low | Low | Moderate | Low | Moderate | Moderate |
| X Yan 2025 | Moderate | Low | Low | Low | Moderate | Low | Low | Moderate |
| X.Sheng 2021 | High | Low | Low | Low | Moderate | Low | Low | High |

**Supplementary Table 4. GRADE evidence assessment.**

| **OS** | | | | | | | | | | | |
| --- | --- | --- | --- | --- | --- | --- | --- | --- | --- | --- | --- |
|  | **Quality assessment** | | | | | | | **No. of patients** | | **Effect** | **Quality** |
| **Comparison** | **No. of studies** | **Design** | **Risk of bias** | **Inconsistency** | **Indirectness** | **Imprecision** | **Other considerations** | **Intervention-1** | **Intervention-2** | **HR (95% CI)** |  |
| **EV vs TPC** | 2 | randomized trials | no serious risk of bias | no serious inconsistency | no serious indirectness | no serious imprecision | undetected | 337 | 1157 | 0.63 (0.43, 0.92) | ⊕⊕⊕⊕ HIGH |
| **EV+Pem vs TPC** | 1 | randomized trials | no serious risk of bias | no serious inconsistency | no serious indirectness | no serious imprecision | undetected | 442 | 1157 | 0.51 (0.34, 0.76) | ⊕⊕⊕⊕ HIGH |
| **SG vs TPC** | 1 | randomized trials | no serious risk of bias | no serious inconsistency | no serious indirectness | serious | undetected | 335 | 1157 | 0.86 (0.58, 1.28) | ⊕⊕⊕O MODERATE |
| **EV vs EV+Pem** | 0 | randomized trials | no serious risk of bias | no serious inconsistency | no serious indirectness | serious | undetected | / | / | 1.24 (0.72, 2.15) | ⊕⊕⊕O MODERATE |
| **EV vs SG** | 0 | randomized trials | no serious risk of bias | no serious inconsistency | no serious indirectness | serious | undetected | / | / | 0.74 (0.43, 1.27) | ⊕⊕⊕O MODERATE |
| **EV+Pem vs SG** | 0 | randomized trials | no serious risk of bias | no serious inconsistency | no serious indirectness | serious | undetected | / | / | 0.59 (0.34, 1.04) | ⊕⊕⊕O MODERATE |
| **ORR** | | | | | | | | | | | |
|  | **Quality assessment** | | | | | | | **No. of patients** | | **Effect** | **Quality** |
| **Comparison** | **No. of studies** | **Design** | **Risk of bias** | **Inconsistency** | **Indirectness** | **Imprecision** | **Other considerations** | **Intervention-1** | **Intervention-2** | **OR (95% CI)** |  |
| **EV vs TPC** | 2 | randomized trials | no serious risk of bias | no serious inconsistency | no serious indirectness | no serious imprecision | undetected | 410 | 1157 | 2.17 (1.15, 4.12) | ⊕⊕⊕⊕ HIGH |
| **EV+Pem vs TPC** | 1 | randomized trials | no serious risk of bias | no serious inconsistency | no serious indirectness | no serious imprecision | undetected | 518 | 1157 | 3.33 (1.65, 6.74) | ⊕⊕⊕⊕ HIGH |
| **SG vs TPC** | 1 | randomized trials | no serious risk of bias | no serious inconsistency | no serious indirectness | serious | undetected | 355 | 1157 | 1.82 (0.75, 4.45) | ⊕⊕⊕O MODERATE |
| **EV vs EV+Pem** | 1 | randomized trials | no serious risk of bias | no serious inconsistency | no serious indirectness | serious | undetected | 410 | 518 | 0.65 (0.30, 1.40) | ⊕⊕⊕O MODERATE |
| **EV vs SG** | 0 | randomized trials | no serious risk of bias | no serious inconsistency | serious | serious | undetected | / | / | 1.19 (0.40, 3.57) | ⊕⊕OO LOW |
| **EV+Pem vs SG** | 0 | randomized trials | no serious risk of bias | no serious inconsistency | serious | serious | undetected | / | / | 1.83 (0.59. 5.70) | ⊕⊕OO LOW |
| **PFS** | | | | | | | | | | | |
|  | **Quality assessment** | | | | | | | **No. of patients** | | **Effect** | **Quality** |
| **Comparison** | **No. of studies** | **Design** | **Risk of bias** | **Inconsistency** | **Indirectness** | **Imprecision** | **Other considerations** | **Intervention-1** | **Intervention-2** | **HR (95% CI)** |  |
| **EV vs TPC** | 2 | randomized trials | no serious risk of bias | no serious inconsistency | no serious indirectness | no serious imprecision | undetected | 337 | 1157 | 0.60 (0.50, 0.72) | ⊕⊕⊕⊕ HIGH |
| **EV+Pem vs TPC** | 1 | randomized trials | no serious risk of bias | no serious inconsistency | no serious indirectness | no serious imprecision | undetected | 442 | 1157 | 0.48 (0.41, 0.57) | ⊕⊕⊕⊕ HIGH |
| **SG vs TPC** | 1 | randomized trials | no serious risk of bias | no serious inconsistency | no serious indirectness | serious | undetected | 335 | 1157 | 0.86 (0.72, 1.03)) | ⊕⊕⊕O MODERATE |
| **EV vs EV+Pem** | 0 | randomized trials | no serious risk of bias | no serious inconsistency | serious | serious | undetected | / | / | 1.26 (0.98, 1.61) | ⊕⊕OO LOW |
| **EV vs SG** | 0 | randomized trials | no serious risk of bias | no serious inconsistency | serious | no serious imprecision | undetected | / | / | 0.70 (0.54, 0.91) | ⊕⊕⊕O MODERATE |
| **EV+Pem vs SG** | 0 | randomized trials | no serious risk of bias | no serious inconsistency | serious | no serious imprecision | undetected | / | / | 0.56 (0.44, 0.71) | ⊕⊕⊕O MODERATE |
| **DCR** | | | | | | | | | | | |
|  | **Quality assessment** | | | | | | | **No. of patients** | | **Effect** | **Quality** |
| **Comparison** | **No. of studies** | **Design** | **Risk of bias** | **Inconsistency** | **Indirectness** | **Imprecision** | **Other considerations** | **Intervention-1** | **Intervention-2** | **OR (95% CI)** |  |
| **EV vs TPC** | 2 | randomized trials | no serious risk of bias | no serious inconsistency | no serious indirectness | no serious imprecision | undetected | 410 | 713 | 2.40 (1.75, 3.30) | ⊕⊕⊕⊕ HIGH |
| **EV vs EV+Pem** | 1 | randomized trials | no serious risk of bias | no serious inconsistency | no serious indirectness | serious | undetected | 410 | 76 | 0.59 (0.24, 1.40) | ⊕⊕⊕O MODERATE |
| **SG vs TPC** | 1 | randomized trials | no serious risk of bias | no serious inconsistency | no serious indirectness | serious | undetected | 355 | 713 | 1.17 (0.86, 1.58) | ⊕⊕⊕O MODERATE |
| **EV+Pem vs TPC** | 0 | randomized trials | no serious risk of bias | no serious inconsistency | no serious indirectness | very serious | undetected | / | / | 4.10 (1.62, 10.40) | ⊕⊕OO LOW |
| **EV vs SG** | 0 | randomized trials | no serious risk of bias | no serious inconsistency | serious | no serious indirectness | undetected | / | / | 2.06 (1.33, 3.20) | ⊕⊕⊕O MODERATE |
| **EV+Pem vs SG** | 0 | randomized trials | no serious risk of bias | no serious inconsistency | serious | serious | undetected | / | / | 3.52 (1.32, 9.37) | ⊕⊕OO LOW |
| **≥3 AES** | | | | | | | | | | | |
|  | **Quality assessment** | | | | | | | **No. of patients** | | **Effect** | **Quality** |
| **Comparison** | **No. of studies** | **Design** | **Risk of bias** | **Inconsistency** | **Indirectness** | **Imprecision** | **Other considerations** | **Intervention-1** | **Intervention-2** | **OR (95% CI)** |  |
| **EV vs TPC** | 2 | randomized trials | no serious risk of bias | no serious inconsistency | no serious indirectness | serious | undetected | 410 | 1157 | 0.81 (0.23, 2.83) | ⊕⊕⊕O MODERATE |
| **EV+Pem vs TPC** | 1 | randomized trials | no serious risk of bias | no serious inconsistency | no serious indirectness | serious | undetected | 518 | 1157 | 1.09 (0.25, 4.85) | ⊕⊕⊕O MODERATE |
| **SG vs TPC** | 1 | randomized trials | no serious risk of bias | serious inconsistency | very serious | serious | undetected | 355 | 1157 | 3.80 (0.59, 24.42) | ⊕OOO VERY LOW |
| **EV vs EV+Pem** | 1 | randomized trials | no serious risk of bias | no serious inconsistency | no serious indirectness | serious | undetected | 410 | 518 | 0.74 (0.16, 3.50) | ⊕⊕⊕O MODERATE |
| **EV vs SG** | 0 | randomized trials | no serious risk of bias | no serious inconsistency | serious | serious | undetected | / | / | 0.21 (0.02, 2.00) | ⊕⊕OO LOW |
| **EV+Pem vs SG** | 0 | randomized trials | no serious risk of bias | no serious inconsistency | serious | serious | undetected | / | / | 0.29 (0.03, 3.11) | ⊕⊕OO LOW |

OS: overall survival; ORR: objective response rate; PFS: progression free survival; DCR: disease control rate; AES: adverse events; TPC: treatment of physician's choice; SG: sacituzumab govitecan; EV: enfortumab vedotin; Pem: pembrolizumab.

**Supplementary Table 5. Network meta-regression results.**

| **Category** | **Mean age** | **Follow-up duration** | **Prior therapy** |
| --- | --- | --- | --- |
| **OS** | -0.01 (-1.84, 3.95) | 0.55 (-3.31, 8.02) | -0.28 (-1.36, 0.70) |
| **ORR** | -0.20 (-1.55, 1.15) | -0.37 (-2.63, 1.71) | -0.28 (-1.71, 1.22) |
| **PFS** | -0.09 (-1.47, 2.4236) | 0.30 (-2.73, 5.10) | -0.12 (-0.86, 0.59) |
| **DCR** | -0.22 (-7.65, 0.47) | -0.03 (-5.50, 4.73) | -0.11 (-1.35, 1.07) |
| **≥3 AES** | -0.27 (-1.99, 1.51) | -1.34 (-3.69, 0.98) | -0.45 (-2.21, 1.36) |

OS: overall survival; ORR: objective response rate; PFS: progression free survival; DCR: disease control rate; AES: adverse events.

**Supplementary Table 6. Regression analysis for single-arm studies.**

| **Subgroup** | **Categories** | **OS** | | | **ORR** | | | **PFS** | | | **DCR** | | | **≥3 AES** | | |
| --- | --- | --- | --- | --- | --- | --- | --- | --- | --- | --- | --- | --- | --- | --- | --- | --- |
|  |  | **Coeff** | **SE** | ***P*** | **Coeff** | **SE** | ***P*** | **Coeff** | **SE** | ***P*** | **Coeff** | **SE** | ***P*** | **Coeff** | **SE** | ***P*** |
| Molecular | HER2- | Ref | Ref | Ref | Ref | Ref | Ref | Ref | Ref | Ref | Ref | Ref | Ref | Ref | Ref | Ref |
|  | HER2+ | -0.16 | 0.52 | 0.751 | 0.13 | 0.58 | 0.819 | 0.28 | 0.17 | 0.098 | -0.52 | 1.10 | 0.634 | 0.52 | 1.73 | 0.764 |
|  | Unspecified | 0.02 | 0.25 | 0.924 | 0.20 | 0.31 | 0.527 | 0.06 | 0.11 | 0.576 | -0.68 | 0.47 | 0.145 | -0.48 | 0.76 | 0.529 |
| Age | <70 | Ref | Ref | Ref | Ref | Ref | Ref | Ref | Ref | Ref | Ref | Ref | Ref | Ref | Ref | Ref |
|  | ≥70 | 0.00 | 0.33 | 0.997 | 0.47 | 0.35 | 0.182 | -0.05 | 0.21 | 0.810 | 0.39 | 0.55 | 0.473 | 0.16 | 0.91 | 0.860 |
| Prior | ≥1 line | Ref | Ref | Ref | Ref | Ref | Ref | Ref | Ref | Ref | Ref | Ref | Ref | Ref | Ref | Ref |
|  | ≥2 lines | 0.27 | 0.52 | 0.609 | -0.07 | 0.49 | 0.879 | -0.19 | 0.19 | 0.307 | 1.59 | 0.69 | **0.021** | -1.72 | 1.61 | 0.285 |
|  | Prior ADC/ICI | -0.27 | 0.28 | 0.331 | -0.51 | 0.37 | 0.169 | -0.23 | 0.12 | 0.056 | -0.07 | 0.56 | 0.904 | -0.55 | 0.85 | 0.520 |
|  | PD after therapy | 0.26 | 0.26 | 0.318 | 0.42 | 0.34 | 0.226 | 0.01 | 0.13 | 0.952 | 0.46 | 0.49 | 0.352 | 1.45 | 0.81 | 0.073 |
|  | Prior untreated | / | 0.24 | 0.318 | -0.23 | 0.61 | 0.704 | 0.44 | 2.21 | 1.039 | 0.03 | -0.19 | 1.166 | 0.87 | / | / |
| Country | America | Ref | Ref | Ref | Ref | Ref | Ref | Ref | Ref | Ref | Ref | Ref | Ref | Ref | Ref | Ref |
|  | British | / | / | / | 0.63 | 0.8 | 0.436 | / | / | / | 0.82 | 0.87 | 0.346 | / | / | / |
|  | China | 0.12 | 0.24 | 0.623 | 0.06 | 0.3 | 0.844 | 0.14 | 0.1 | 0.188 | 0.99 | 0.34 | **0.003** | -0.52 | 0.76 | 0.489 |
|  | France | / | / | / | 0.05 | 0.78 | 0.950 | / | / | / | -1.65 | 0.81 | **0.042** | / | / | / |
|  | Japan | / | / | / | -0.59 | 0.57 | 0.301 | 0.33 | 0.3 | 0.270 | 0.52 | 0.61 | 0.396 | 0.08 | 1.13 | 0.941 |
|  | Netherlands | -0.59 | 0.5 | 0.240 | -0.44 | 0.88 | 0.618 | -0.97 | 0.37 | **0.009** | -1.22 | 0.89 | 0.167 | -0.15 | 1.76 | 0.934 |
| Time | <12 | Ref | Ref | Ref | Ref | Ref | Ref | Ref | Ref | Ref | Ref | Ref | Ref | Ref | Ref | Ref |
|  | ≥12 | 0.40 | 0.22 | 0.065 | 0.42 | 0.29 | 0.146 | 0.18 | 0.11 | 0.092 | 0.89 | 0.41 | **0.030** | -2.02 | 0.70 | **0.004** |

OS: overall survival; ORR: objective response rate; PFS: progression free survival; DCR: disease control rate; AES: adverse events; PD: progressive disease; ADC: antibody-drug conjugates; ICI: immune checkpoint inhibitors. The bolded *P* value indicates statistical significance.

**Supplementary Table 7. Subgroup analysis of single-arm studies.**

| **Subgroups** | **Categories** | **OS** | | | | | **ORR** | | | | | **PFS** | | | | | **DCR** | | | | | **≥3 AES** | | | | |
| --- | --- | --- | --- | --- | --- | --- | --- | --- | --- | --- | --- | --- | --- | --- | --- | --- | --- | --- | --- | --- | --- | --- | --- | --- | --- | --- |
|  |  | **N** | **mOS** | **95%CI** | **I^2^** | ***P*** | **N** | **P-pooled** | **95%CI** | **I^2^** | ***P*** | **N** | **mPFS** | **95%CI** | **I^2^** | ***P*** | **N** | **P-pooled** | **95%CI** | **I^2^** | ***P*** | **N** | **P-pooled** | **95%CI** | **I^2^** | ***P*** |
| Molecular | HER2- | 6 | 13.06 | 7.97-21.41 | 95 | 0.830 | 10 | 0.46 | 0.37-0.56 | 50 | 0.824 | 7 | 5.83 | 4.63-7.35 | 75 | **<0.001** | 8 | 0.85 | 0.72-0.93 | 65 | 0.384 | 7 | 0.59 | 0.27-0.85 | 72 | 0.249 |
|  | HER2+ | 1 | 11.00 | 6.44-18.79 | / |  | 2 | 0.49 | 0.33-0.66 | 73 |  | 2 | 7.99 | 7.51-8.51 | 0 |  | 1 | 0.77 | 0.58-0.90 | / |  | 1 | 0.70 | 0.51-0.85 | / |  |
|  | Unspecified | 6 | 13.01 | 11.70-14.81 | 23 |  | 20 | 0.51 | 0.41-0.60 | 76 |  | 11 | 5.81 | 5.38-6.28 | 27 |  | 19 | 0.75 | 0.64-0.83 | 73 |  | 15 | 0.47 | 0.28-0.67 | 90 |  |
| Age | <70 | 11 | 12.98 | 10.02-16.81 | 91 | 0.723 | 27 | 0.46 | 0.39-0.53 | 0.12 | 0.358 | 18 | 6.30 | 5.62-7.07 | 83 | 0.767 | 23 | 0.76 | 0.66-0.84 | 78 | 0.945 | 19 | 0.51 | 0.32-0.70 | 88 | 0.740 |
|  | ≥70 | 2 | 13.84 | 10.84-17.67 | 0 |  | 5 | 0.56 | 0.44-0.67 | 50 |  | 2 | 5.88 | 4.63-7.47 | 0 |  | 5 | 0.74 | 0.67-0.80 | 30 |  | 4 | 0.56 | 0.33-0.78 | 76 |  |
| Piror | ≥1 line | 5 | 13.67 | 11.70-15.97 | 42 | 0.320 | 16 | 0.49 | 0.40-0.58 | 67 | 0.162 | 10 | 6.61 | 5.58-7.82 | 81 | **0.039** | 14 | 0.72 | 0.57-0.83 | 81 | **0.001** | 11 | 0.48 | 0.32-0.65 | 79 | 0.086 |
|  | ≥2 lines | 1 | 16.40 | 8.26-32.56 | / |  | 3 | 0.48 | 0.32-0.64 | 51 |  | 1 | 5.50 | 4.55-6.65 | / |  | 4 | 0.90 | 0.79-0.95 | 0 |  | 1 | 0.16 | 0.03-0.4 | / |  |
|  | Piror ADC/ICI | 3 | 9.60 | 6.30-14.62 | 92 |  | 5 | 0.40 | 0.34-0.45 | 0 |  | 3 | 5.34 | 4.89-5.82 | 0 |  | 3 | 0.69 | 0.63-0.74 | 15 |  | 4 | 0.35 | 0.13-0.66 | 95 |  |
|  | PD after therapy | 4 | 16.43 | 10.53-25.65 | 88 |  | 6 | 0.62 | 0.42-0.79 | 87 |  | 5 | 6.65 | 5.74-7.70 | 38 |  | 5 | 0.78 | 0.63-0.89 | 71 |  | 5 | 0.84 | 0.34-0.98 | 90 |  |
|  | Piror untreat | / | / | / | / |  | 2 | 0.46 | 0.15-0.80 | 81 |  | 1 | 8.53 | 5.00-14.56 | / |  | 2 | 0.94 | 0.80-0.99 | 0 |  | 2 | 0.44 | 0.09-0.85 | 90 |  |
| Country | China | 6 | 14.52 | 9.40-22.43 | 96 | 0.127 | 19 | 0.50 | 0.41-0.60 | 71 | 0.421 | 11 | 6.69 | 5.83-7.68 | 83 | **0.002** | 14 | 0.82 | 0.78-0.85 | 45 | **<0.001** | 11 | 0.45 | 0.21-0.72 | 84 | 0.867 |
|  | America | 6 | 12.39 | 10.98-13.98 | 0 |  | 9 | 0.50 | 0.36-0.64 | 79 |  | 7 | 5.58 | 5.13-6.07 | 0 |  | 8 | 0.69 | 0.54-0.81 | 79 |  | 8 | 0.58 | 0.30-0.82 | 93 |  |
|  | Netherlands | 1 | 7.03 | 3.93-12.58 | / |  | 1 | 0.38 | 0.14-0.68 | / |  | 1 | 2.20 | 1.15-4.20 | / |  | 1 | 0.38 | 0.14-0.68 | / |  | 1 | 0.54 | 0.25-0.81 | / |  |
|  | Japan | / | / | / | / |  | 3 | 0.35 | 0.21-0.52 | 0 |  | 1 | 8.10 | 4.97-13.20 | / |  | 3 | 0.76 | 0.60-0.88 | 0 |  | 3 | 0.59 | 0.42-0.74 | 0 |  |
|  | France | / | / | / | / |  | 1 | 0.50 | 0.29-0.71 | / |  | / | / | / | / |  | 1 | 0.29 | 0.13-0.51 | / |  | / | / | / | / |  |
|  | British | / | / | / | / |  | 1 | 0.64 | 0.42-0.56 | / |  | / | / | / | / |  | 1 | 0.82 | 0.60-0.95 | / |  | / | / | / | / |  |
| Time | <12 | 8 | 11.17 | 8.72-14.31 | 91 | 0.072 | 12 | 0.41 | 0.34-0.48 | 58 | <0.001 | 9 | 5.51 | 4.94-6.15 | 49 | 0.058 | 12 | 0.64 | 0.53-0.75 | 80 | 0.003 | 8 | 0.78 | 0.51-0.92 | 82 | 0.030 |
|  | ≥12 | 5 | 16.68 | 11.65-23.87 | 78 |  | 11 | 0.52 | 0.43-0.60 | 64 |  | 11 | 6.64 | 5.77-7.65 | 89 |  | 7 | 0.81 | 0.70-0.89 | 74 |  | 8 | 0.31 | 0.14-0.55 | 91 |  |

OS: overall survival; ORR: objective response rate; PFS: progression free survival; DCR: disease control rate; AES: adverse events; PD: progressive disease; ADC: antibody-drug conjugates; ICI: immunotherapy.

**Supplementary Table 8. Sensitive analysis of NMA.**

**A. Excluding studies with small simple size.**

**a. Overall survival (OS)**

| **EV** |  |  |  |
| --- | --- | --- | --- |
| **1.37 (1.03, 1.83)** | **EV+Pem** |  |  |
| 0.81 (0.61, 1.08) | **0.59 (0.47, 0.76)** | **SG** |  |
| **0.70 (0.56, 0.88)** | **0.51 (0.43, 0.61)** | 0.86 (0.73, 1.02) | **ST** |

**b. Objective response rate (ORR)**

| **EV** |  |  |  |
| --- | --- | --- | --- |
| 1.19 (0.75, 1.89) | **EV+Pem** |  |  |
| **1.71 (1.00, 2.94)** | 1.44 (0.89, 2.31) | **SG** |  |
| **3.12 (2.15, 4.53)** | **2.62 (1.99, 3.44)** | **1.82 (1.23, 2.69)** | **ST** |

**c. Progression free survival (PFS)**

| **EV** |  |  |  |
| --- | --- | --- | --- |
| 1.29 (1.00, 1.66) | **EV+Pem** |  |  |
| **0.72 (0.55, 0.94)** | **0.56 (0.44, 0.71)** | **SG** |  |
| **0.62 (0.51, 0.75)** | **0.48 (0.41, 0.57)** | 0.86 (0.72, 1.03) | **ST** |

**d. Disease control rate (DCR)**

| **EV** |  |  |
| --- | --- | --- |
| **2.11 (1.34, 3.32)** | **SG** |  |
| **2.46 (1.76, 3.44)** | 1.17 (0.86, 1.58) | **ST** |

**e. Grade ≥3 adverse events (AES)**

| **EV** |  |  |  |
| --- | --- | --- | --- |
| **3.10 (1.69, 5.68)** | **EV+Pem** |  |  |
| 0.48 (0.26, 0.89) | **0.15 (0.10, 0.23)** | **SG** |  |
| **1.81 (1.06, 3.11)** | **0.58 (0.44, 0.77)** | **3.80 (2.79, 5.19)** | **ST** |

**B. Excluding non-TPC** **control arms' studies.**

**a. Objective response rate (ORR)**

| **EV** |  |  |  |
| --- | --- | --- | --- |
| 1.08 (0.62, 1.91) | **EV+Pem** |  |  |
| 1.56 (0.83, 2.93) | 1.44 (0.78, 2.64) | **SG** |  |
| **2.84 (1.87, 4.31)** | **2.62 (1.79, 3.84)** | **1.82 (1.14, 2.93)** | **ST** |

**b. Disease control rate (DCR)**

| **EV** |  |  |
| --- | --- | --- |
| **2.06 (1.33, 3.20)** | **SG** |  |
| **2.40 (1.75, 3.30)** | 1.17 (0.86, 1.58) | **ST** |

**c. Grade ≥3 adverse events (AES)**

| EV |  |  |  |
| --- | --- | --- | --- |
| 2.06 (0.47, 9.02) | EV+Pem |  |  |
| 0.32 (0.07, 1.40) | **0.15 (0.03, 0.78)** | SG |  |
| 1.20 (0.47, 3.07) | 0.58 (0.19, 1.83) | **3.80 (1.20, 12.04)** | **ST** |

Comparisons between treatments defined in the columns versus treatments defined in the rows. Statistically significant results are highlighted in bold. Results for survival outcomes, OS and PFS, were presented as hazard ratios (HR) with 95% confidence intervals (CI). Results for dichotomous outcomes, ORR, DCR, AEs, were presented as odds ratios (OR) with 95% CIs. TPC: treatment of physician's choice; SG: sacituzumab govitecan; EV: enfortumab vedotin; Pem: pembrolizumab. OS: overall survival; ORR: objective response rate; PFS: progression free survival; DCR: disease control rate; AES: adverse events.

**Supplementary Figure 1. Risk of bias in randomized controlled trials.**


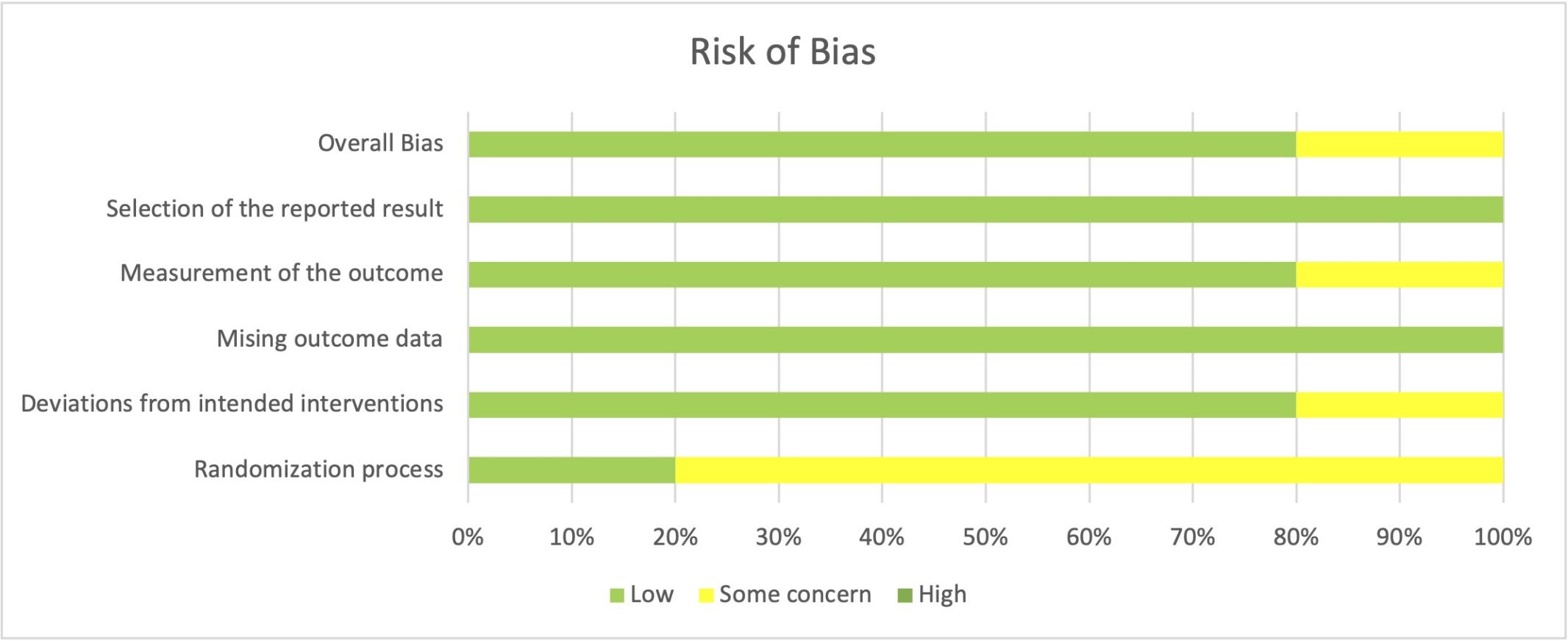


**Supplementary Figure 2. Risk of bias in single-arm studies.**


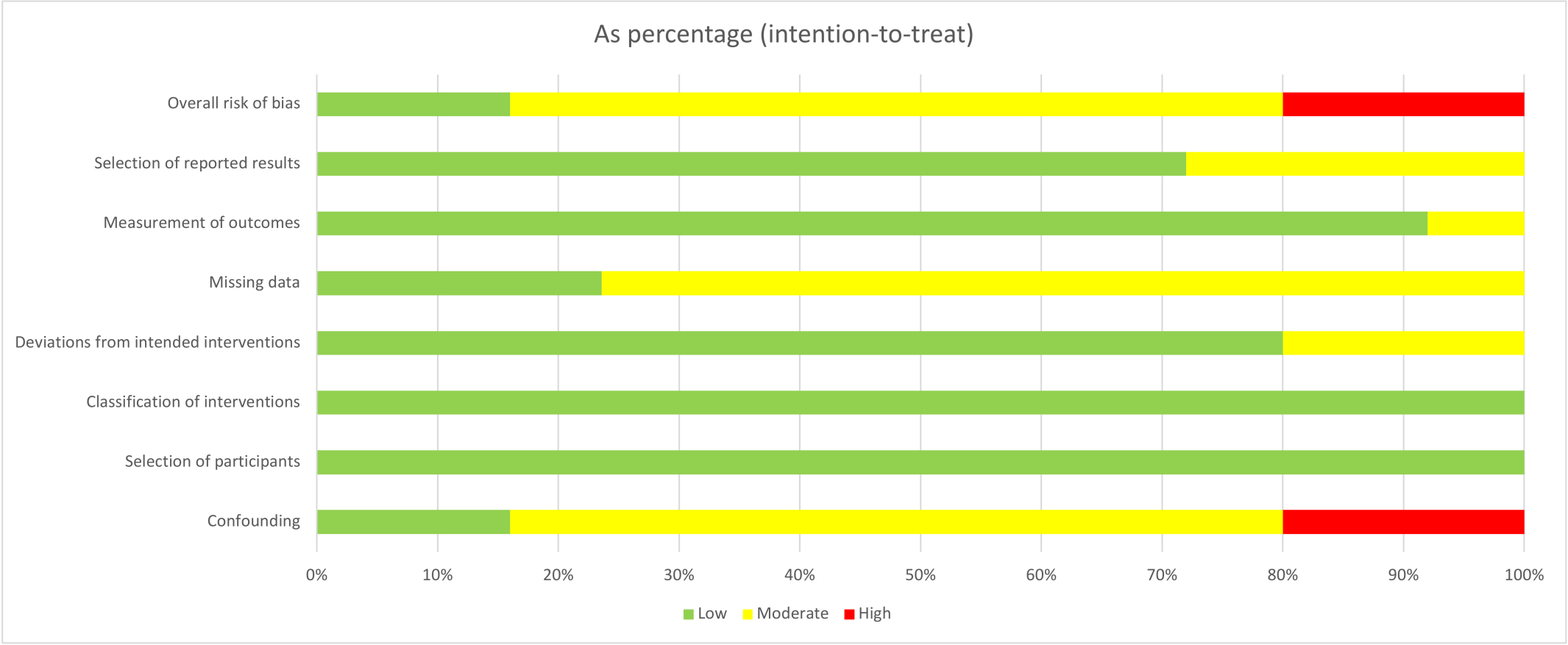


**Supplementary Figure 3. Node splitting results of randomized controlled trials.**

1. **Objective response rate (ORR)**


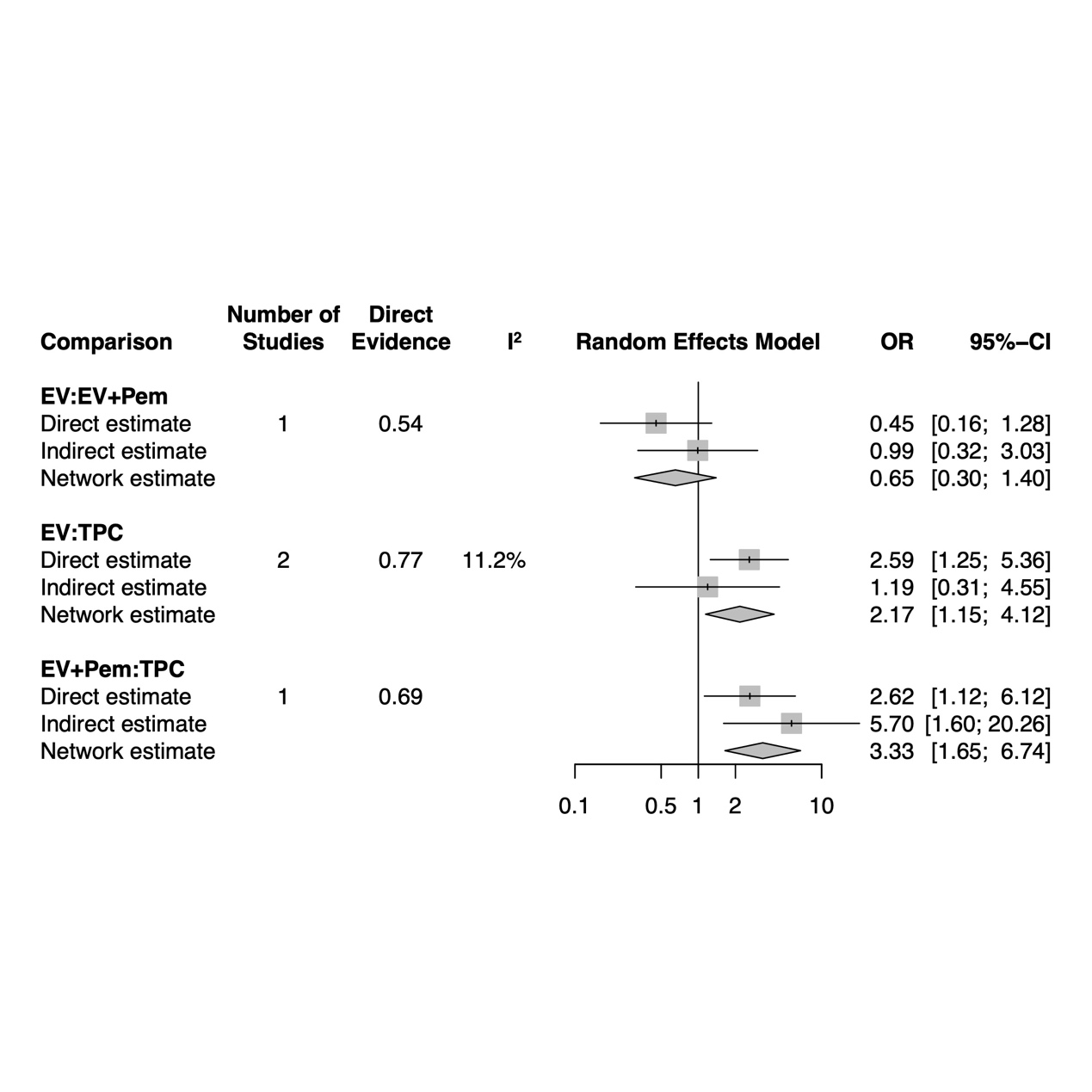


1. **Grade ≥3 adverse events (AEs)**


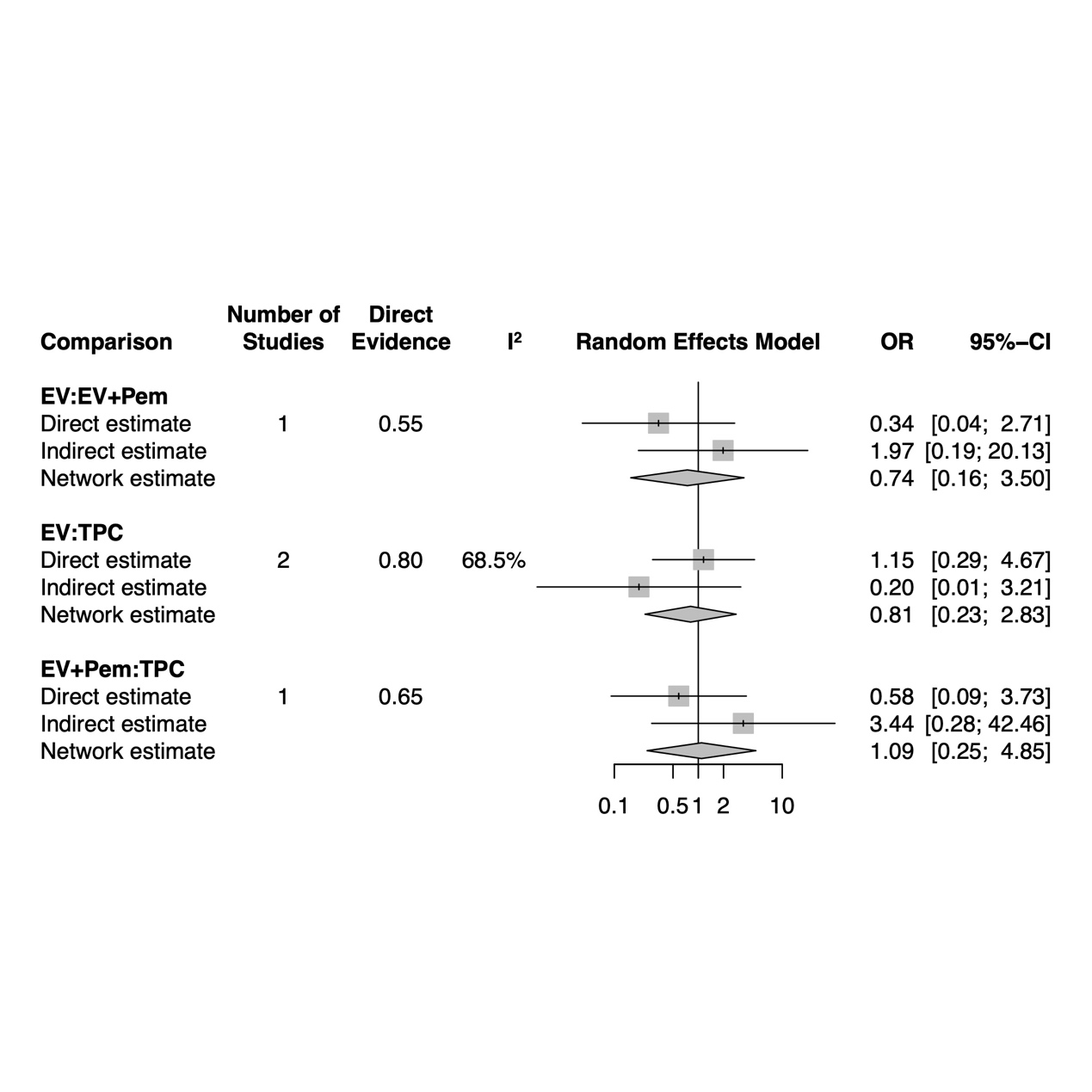


TPC: treatment of physician's choice; EV: enfortumab vedotin; Pem: pembrolizumab.

**Supplementary Figure 4. Forest plots of single-arm trials.**

1. **Median overall survival (mOS)**

1. **Objective response rate (ORR)**

1. **Median progression-free survival (mPFS)**

1. **Disease control rate (DCR)**

1. **Grade ≥3 adverse events (AEs)**

DV: disitamab vedotin; TV: trastuzumab vedotin; SG: sacituzumab govitecan; EV: enfortumab vedotin; TDM-1: trastuzumab emtansine; T-DXd: trastuzumab deruxtecan; Nivo: nivolumab; DV: disitamab vedotin; ICI: immune checkpoint inhibitors; TP: toripalimab; BF: bulumtatug fuvedotin.

**Supplementary Figure 5. Funnel Plots of single-arm trials.**

1. **Median overall survival (mOS)**

**
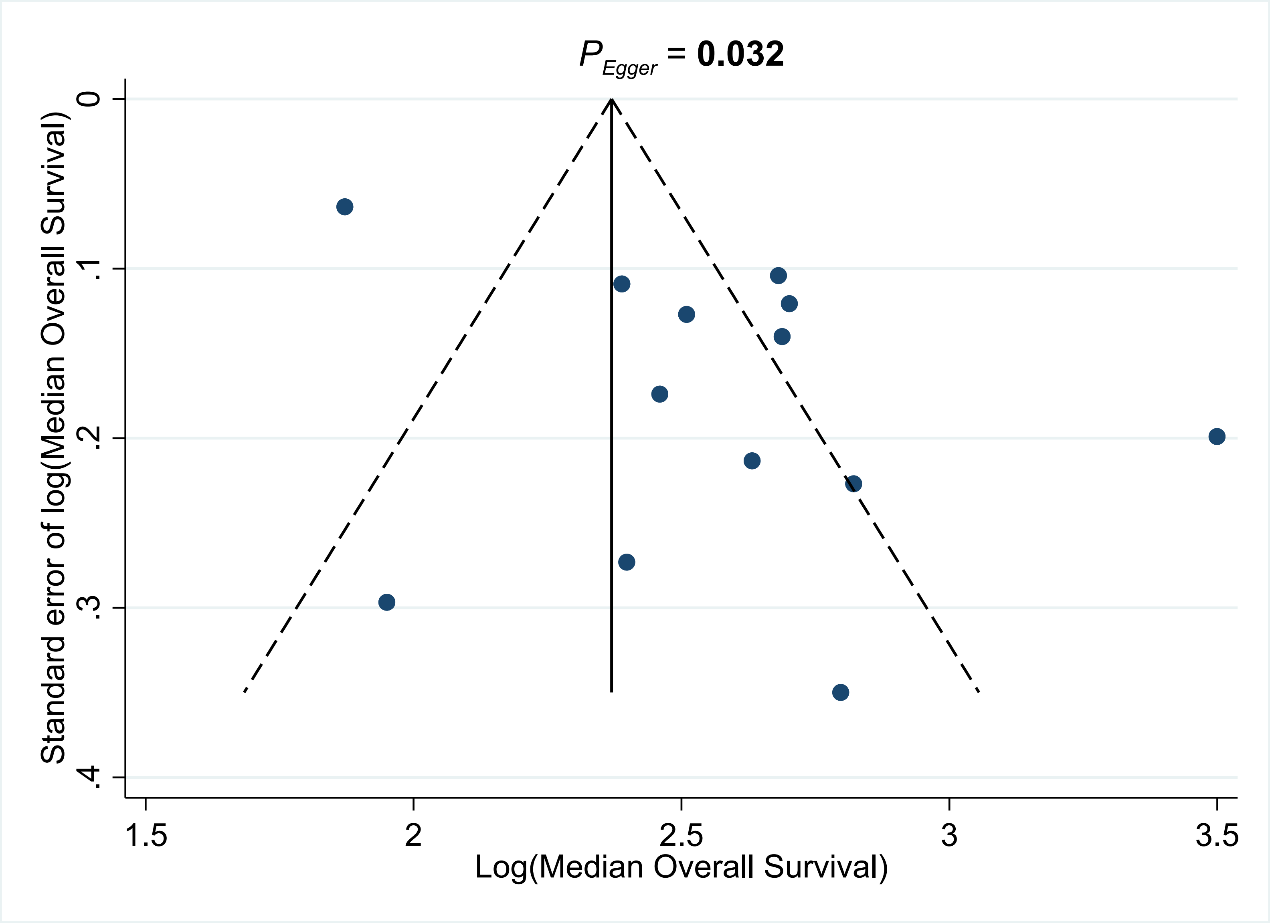
**

1. **Objective response rate (ORR)**


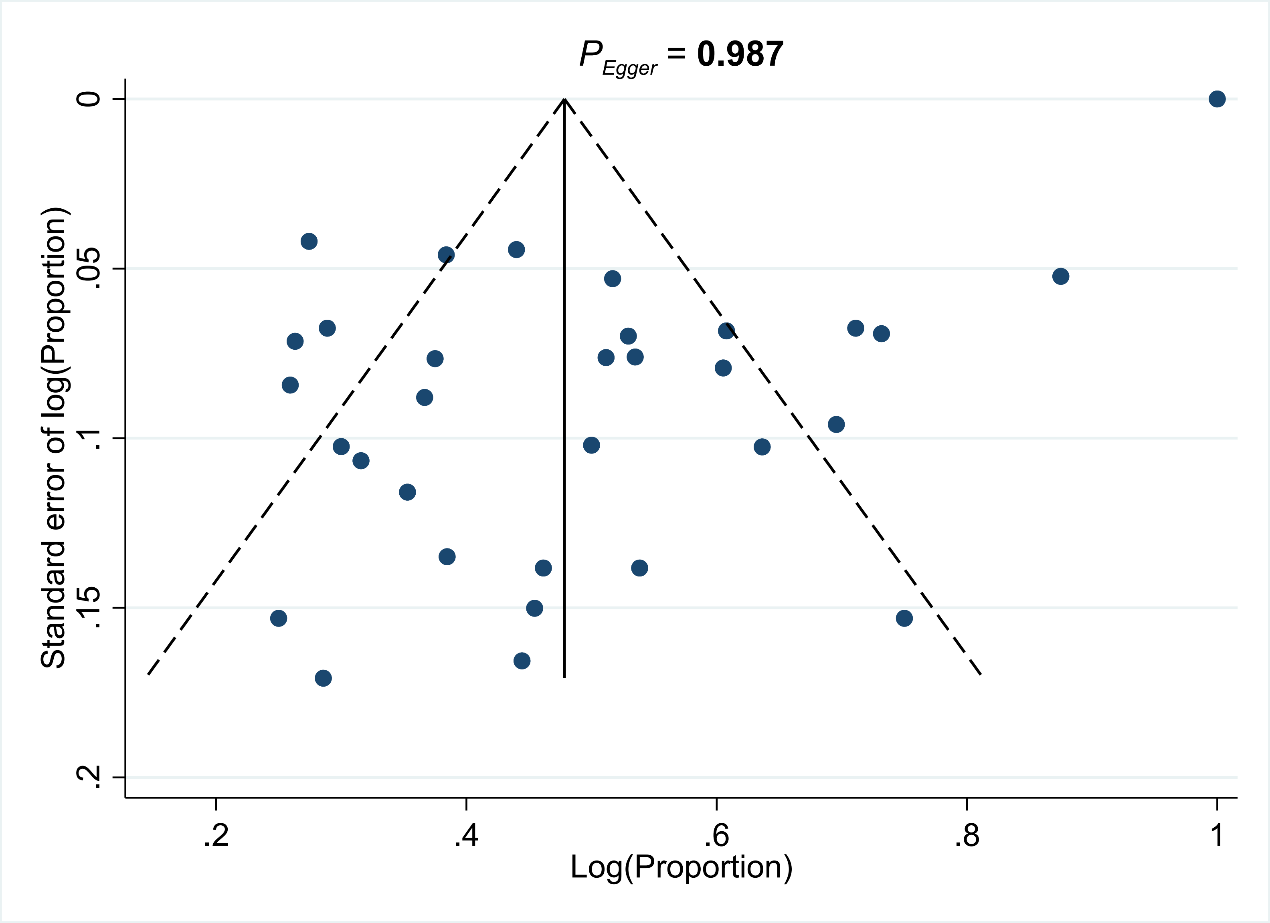


**C. Median progression-free survival (mPFS)**

**
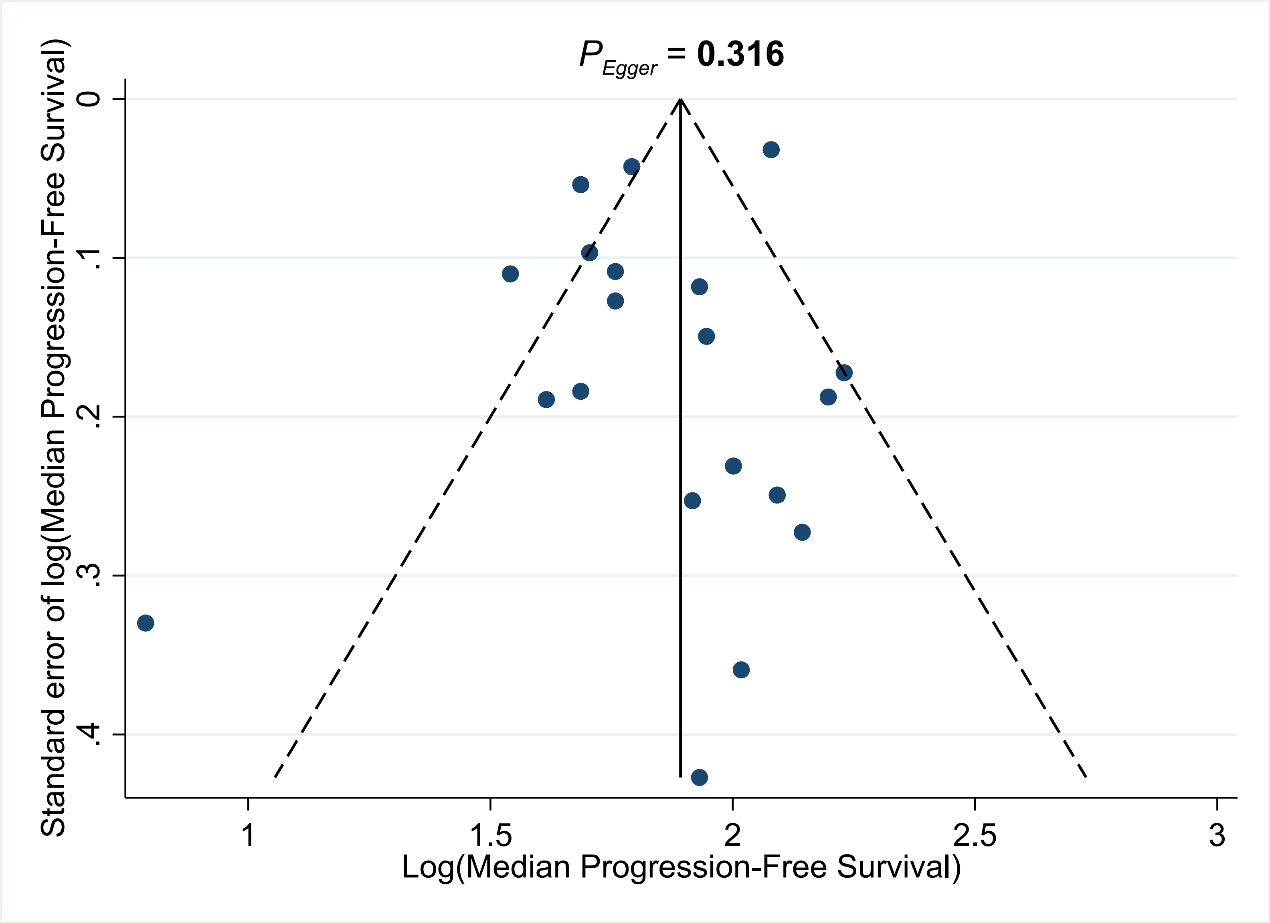
**

**D. Disease control rate (DCR)**


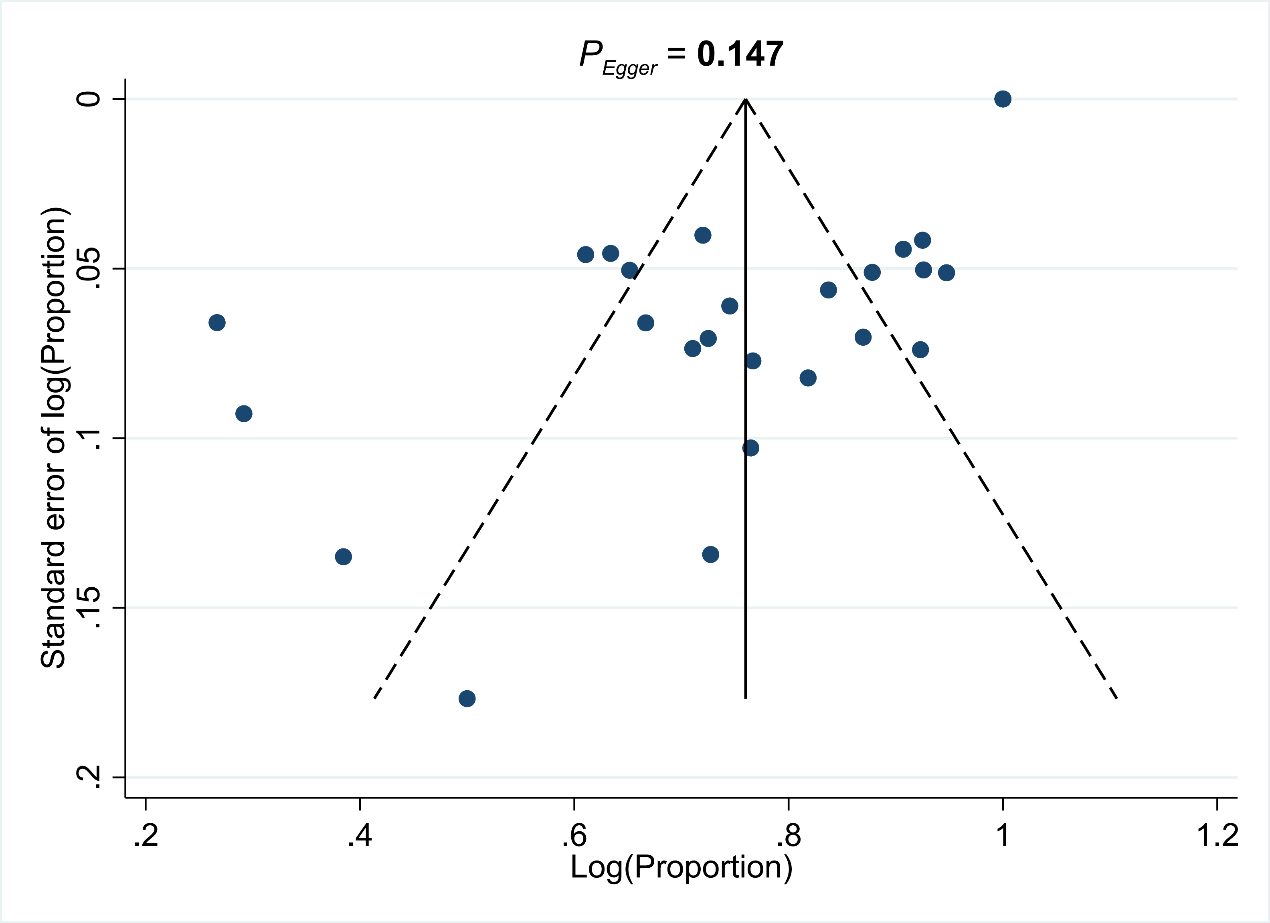


**E. Grade ≥3 adverse events (AEs)**


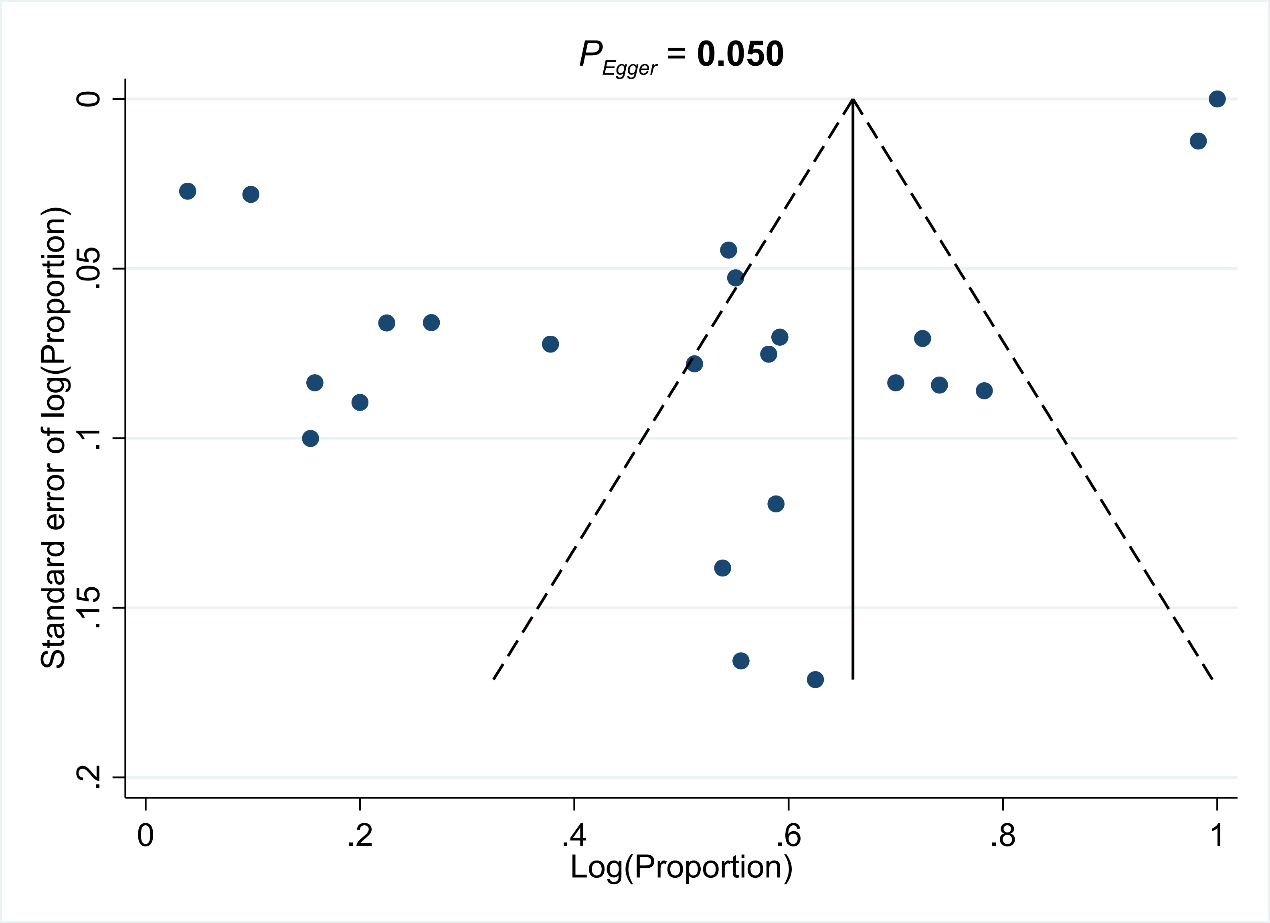


**Supplementary Figure 6. Sensitive analysis of NMA (P-score).**

**A. Excluding studies with small simple size.**

**
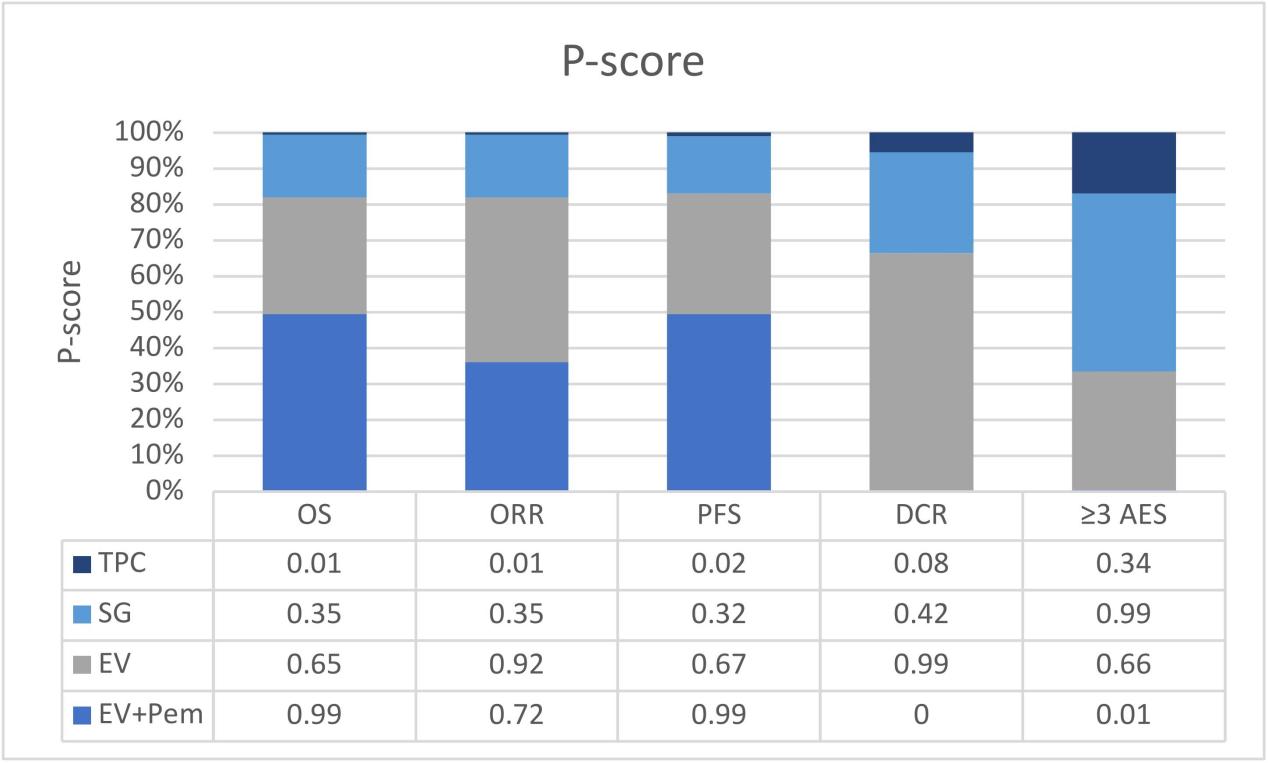
**

**B. Excluding non-TPC** **control arms' studies.
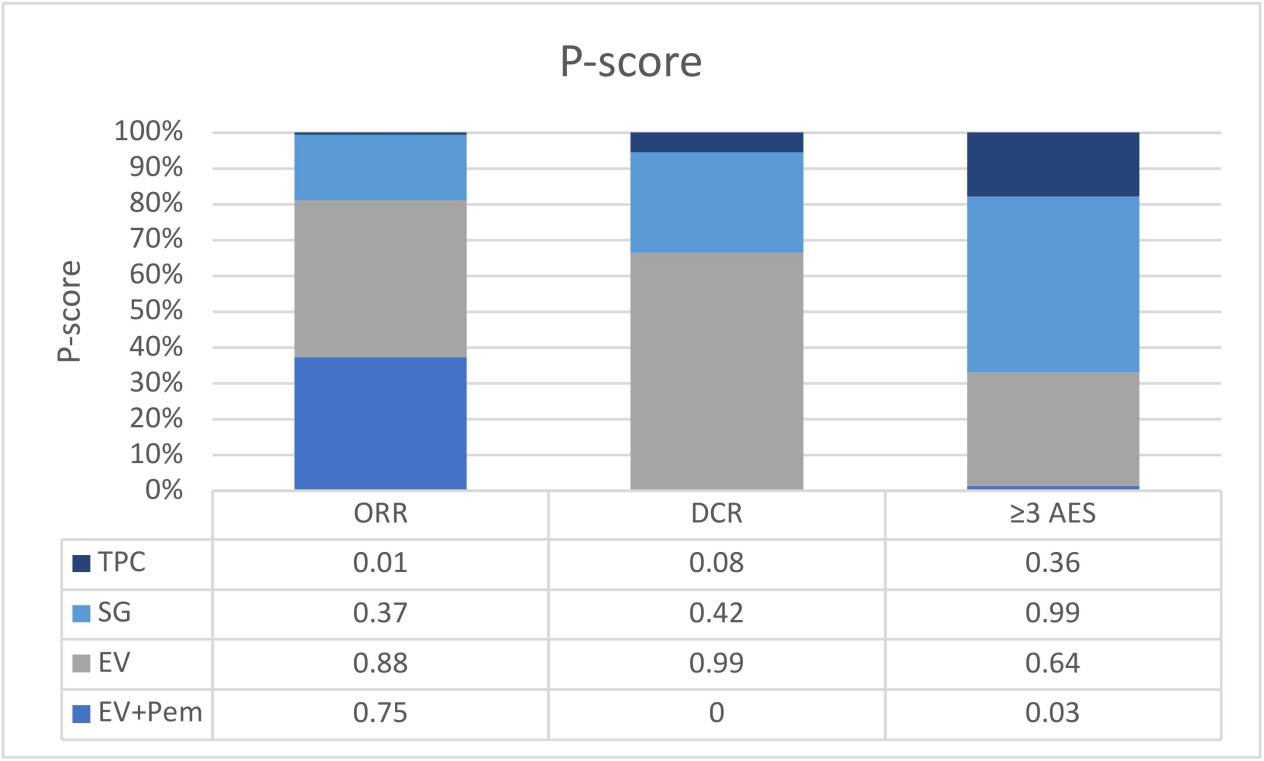
**

OS: overall survival; ORR: objective response rate; PFS: progression free survival; DCR: disease control rate; AES: adverse events; TPC: treatment of physician's choice; SG: sacituzumab govitecan; EV: enfortumab vedotin; Pem: pembrolizumab.

**Supplementary Figure 7. Sensitive analysis of single-arm trials.**

1. **Median overall survival (mOS)**


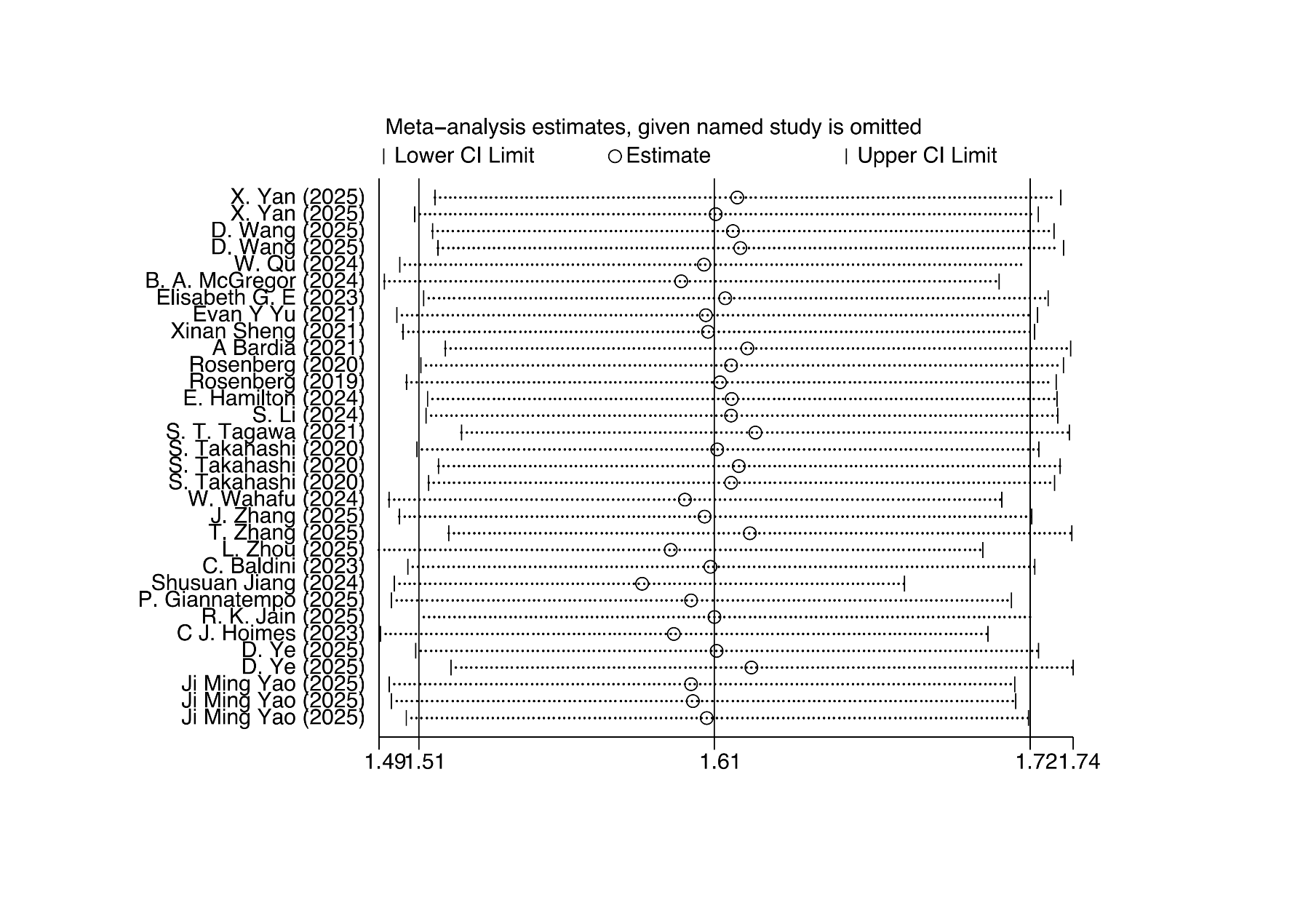


1. **Objective response rate (ORR)**

**
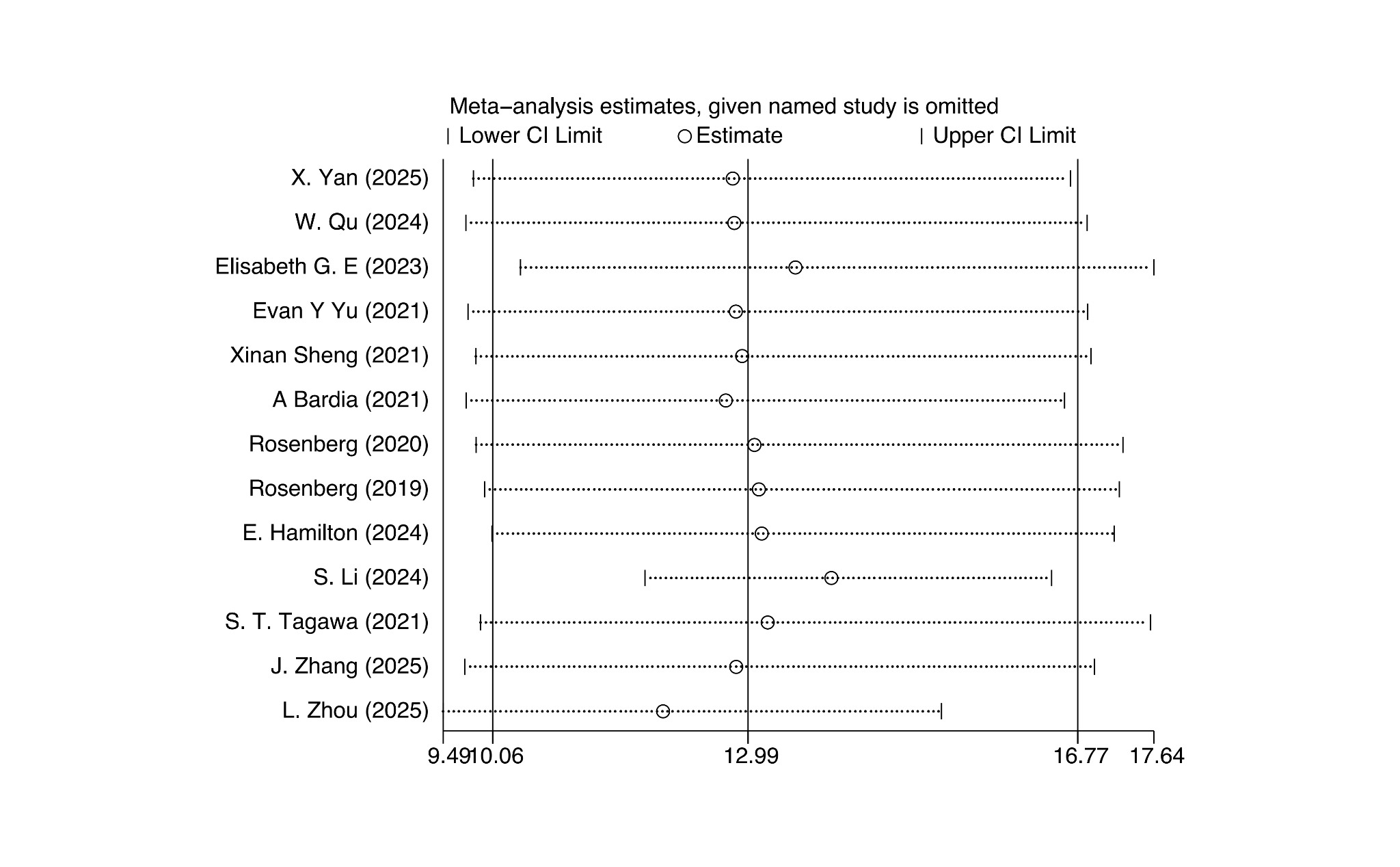
**

1. **Median progression-free survival (mPFS)**


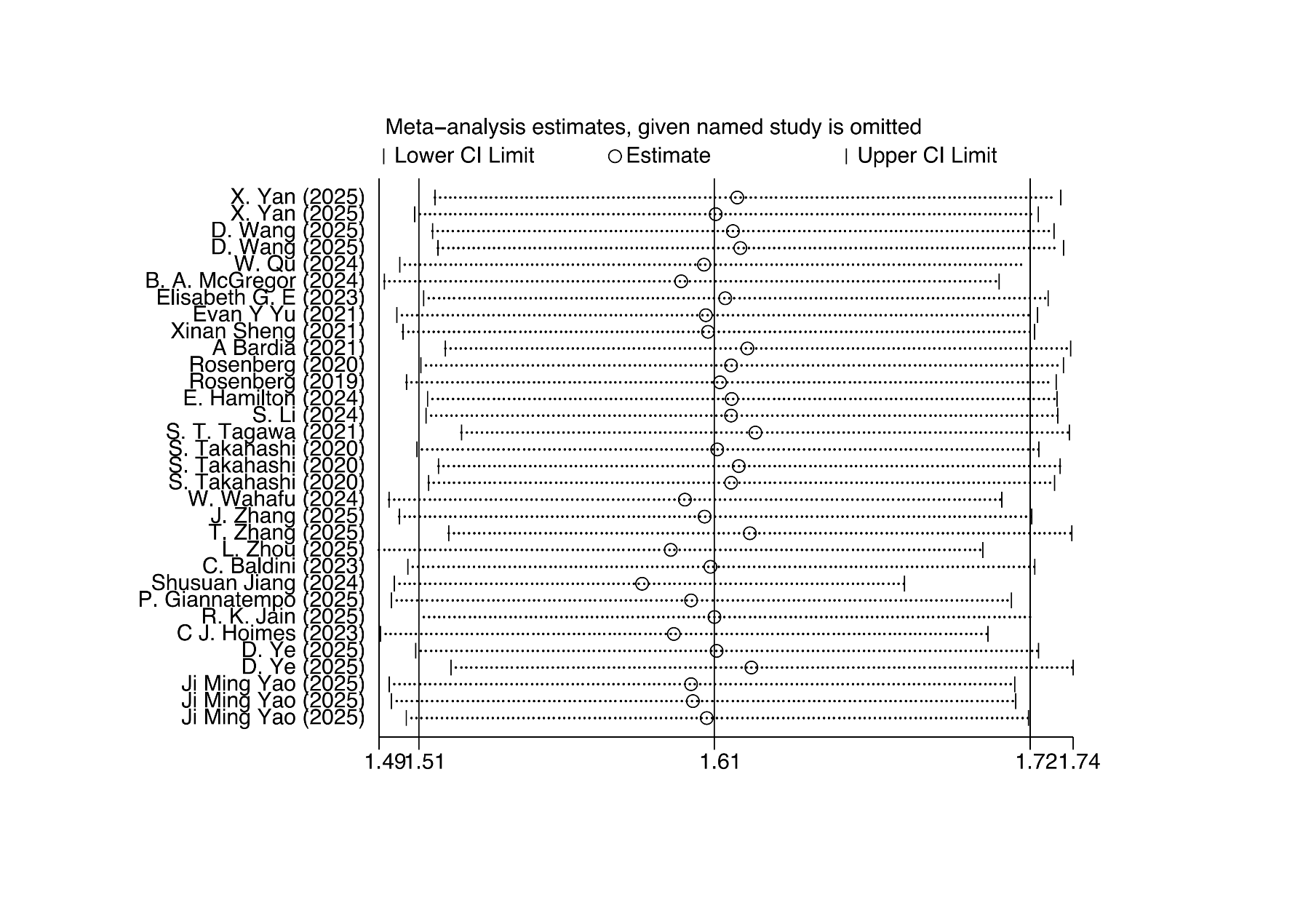


1. **Disease control rate (DCR)**


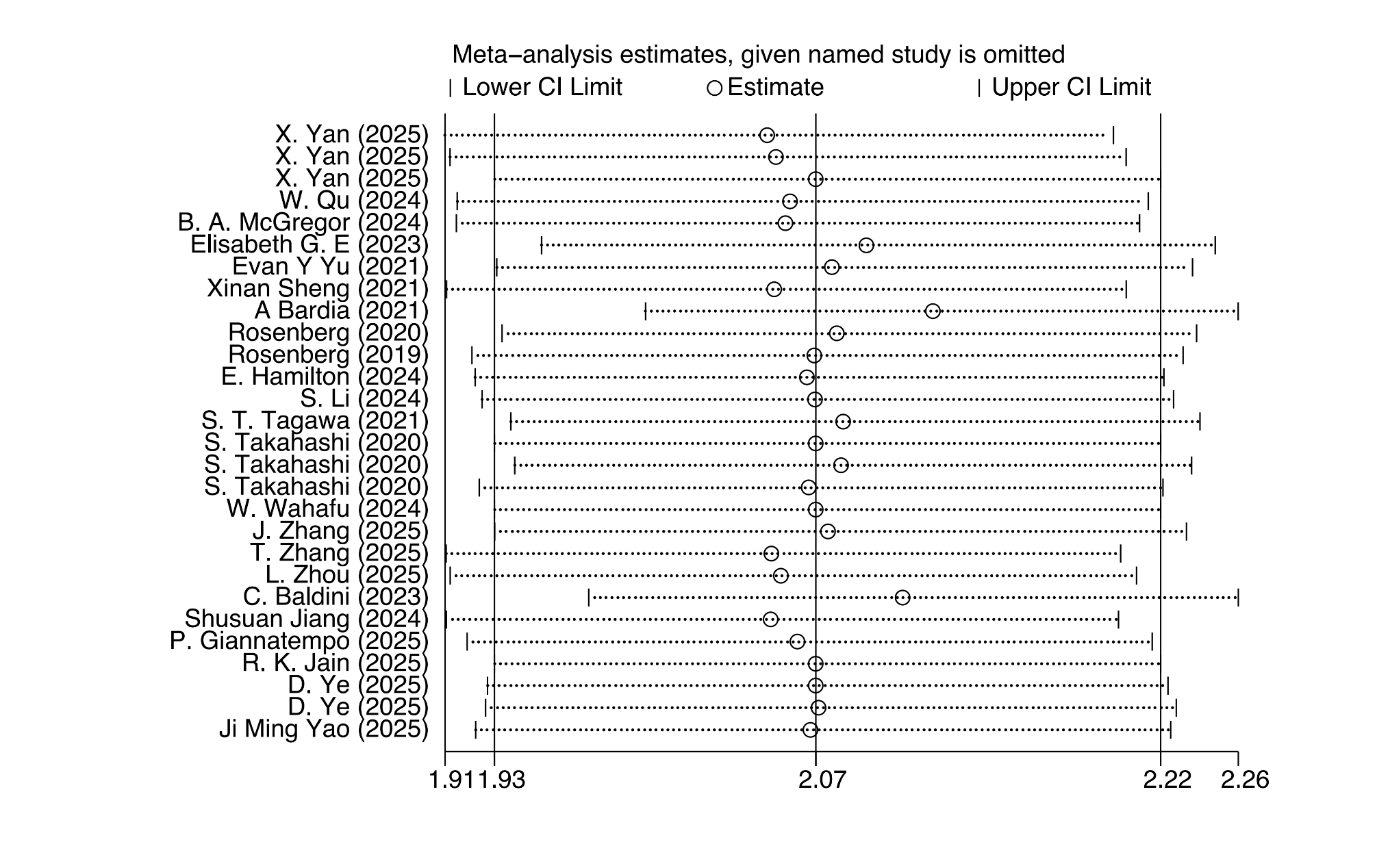


1. **Grade ≥3 adverse events (AEs)**

**
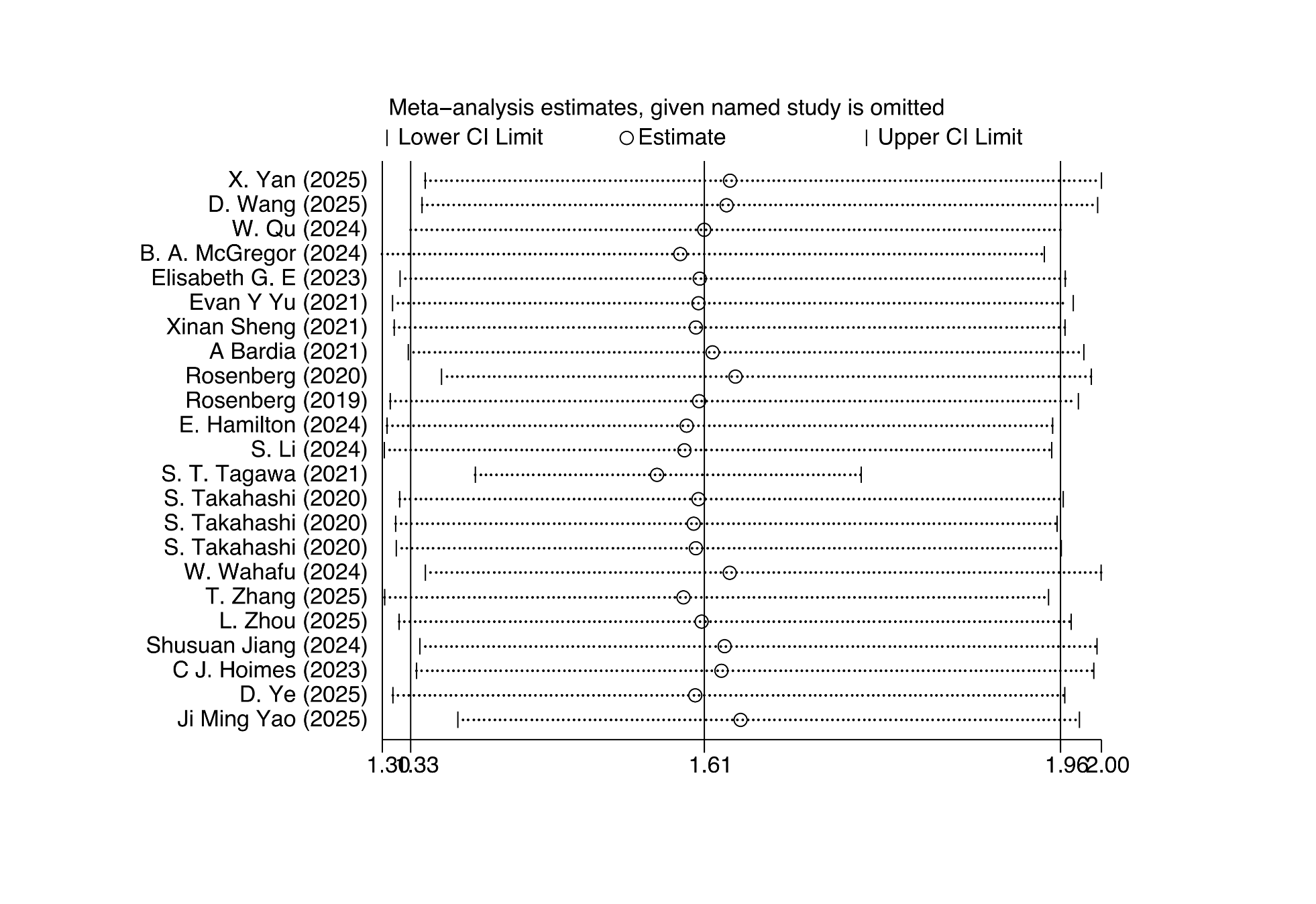
**
